# Dual agonistic and antagonistic roles of ZC3H18 provide for co-activation of distinct nuclear RNA decay pathways

**DOI:** 10.1016/j.celrep.2023.113325

**Published:** 2023-10-26

**Authors:** Patrik Polák, William Garland, Om Rathore, Manfred Schmid, Anna Salerno-Kochan, Lis Jakobsen, Maria Gockert, Piotr Gerlach, Toomas Silla, Jens S. Andersen, Elena Conti, Torben Heick Jensen

**Affiliations:** 1 Department of Molecular Biology and Genetics, Universitetsbyen 81, Aarhus University, Aarhus, Denmark; 2 Department of Structural Cell Biology, Max Planck Institute of Biochemistry, Am Klopferspitz 18, Martinsried/Munich, Germany; 3 Department of Biochemistry and Molecular Biology, University of Southern Denmark, Campusvej 55, Odense M, Denmark

**Keywords:** nuclear RNA decay, activation, inhibition, PAXT, NEXT, ARS2, ZC3H18

## Abstract

The RNA exosome is a versatile ribonuclease. In the nucleoplasm of mammalian cells, it is assisted by its adaptors the nuclear exosome targeting (NEXT) complex and the poly(A) exosome targeting (PAXT) connection. Via its association with the ARS2 and ZC3H18 proteins, NEXT/exosome is recruited to capped and short unadenylated transcripts. Conversely, PAXT/exosome is considered to target longer and adenylated substrates via their poly(A) tails. Here, mutational analysis of the core PAXT component ZFC3H1 uncovers a separate branch of the PAXT pathway, which targets short adenylated RNAs and relies on a direct ARS2-ZFC3H1 interaction. We further demonstrate that similar acidic-rich short linear motifs of ZFC3H1 and ZC3H18 compete for a common ARS2 epitope. Consequently, while promoting NEXT function, ZC3H18 antagonizes PAXT activity. We suggest that this organization of RNA decay complexes provides co-activation of NEXT and PAXT at loci with abundant production of short exosome substrates.

## Highlights

• ZFC3H1 directly interacts with ARS2 to mediate the degradation of short pA^+^ RNAs
• The ZC3H18 protein can antagonize the ARS2-dependent PAXT pathway
• ZFC3H1 and ZC3H18 compete to bind ARS2 via their similar ARMs

## Graphical abstract

**Figure:**
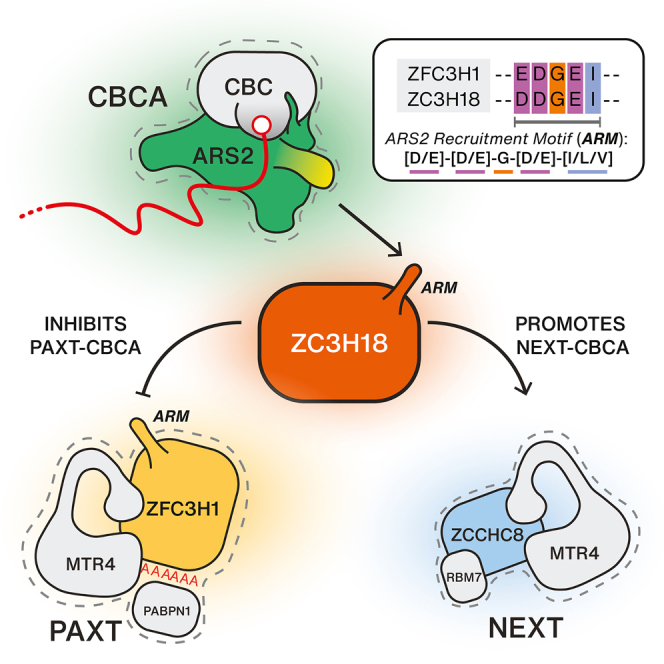

## Teaser

Polák et al. show that the PAXT factor ZFC3H1 interacts with ARS2 via an identified ARS2 recruitment motif (ARM), promoting turnover of short, polyadenylated transcripts. A similar ARM is found in the NEXT-interacting factor ZC3H18, which competes with ZFC3H1 to bind ARS2. This facilitates co-activation of the NEXT and PAXT pathways.

## Introduction

Mammalian genomic DNA is pervasively transcribed by RNA polymerase II (RNAPII), generating a large volume of unstable noncoding RNA (ncRNA) along with the canonical production of mRNA and stable ncRNA.^1^ Relevantly, a large share of RNAPII transcription initiation events is estimated to be non-productive and subjected to premature transcription termination with the released transcript being rapidly removed by RNA decay.^2^^,^^3^^,^^4^ Such termination can occur outside of, or early within, conventional transcription units (TUs) and be induced by the presence of transcription start site (TSS)-proximal polyadenylation (pA) sites,^5^^,^^6^^,^^7^ recruiting the cleavage and pA complex and yielding short pA^+^ RNA.^8^ Features triggering early transcription termination can also be more elusive^9^^,^^10^^,^^11^ and activate the integrator-^12^ or ZC3H4-WDR82 (restrictor) complexes,^13^^,^^14^^,^^15^^,^^16^ giving rise to pA^−^ RNA. Given their essential roles in integral quality control processes that regulate genomic output and maintain transcriptome homeostasis, mechanisms connecting early transcription termination and transcript turnover remain important study objects.^17^^,^^18^

**Figure 1:**
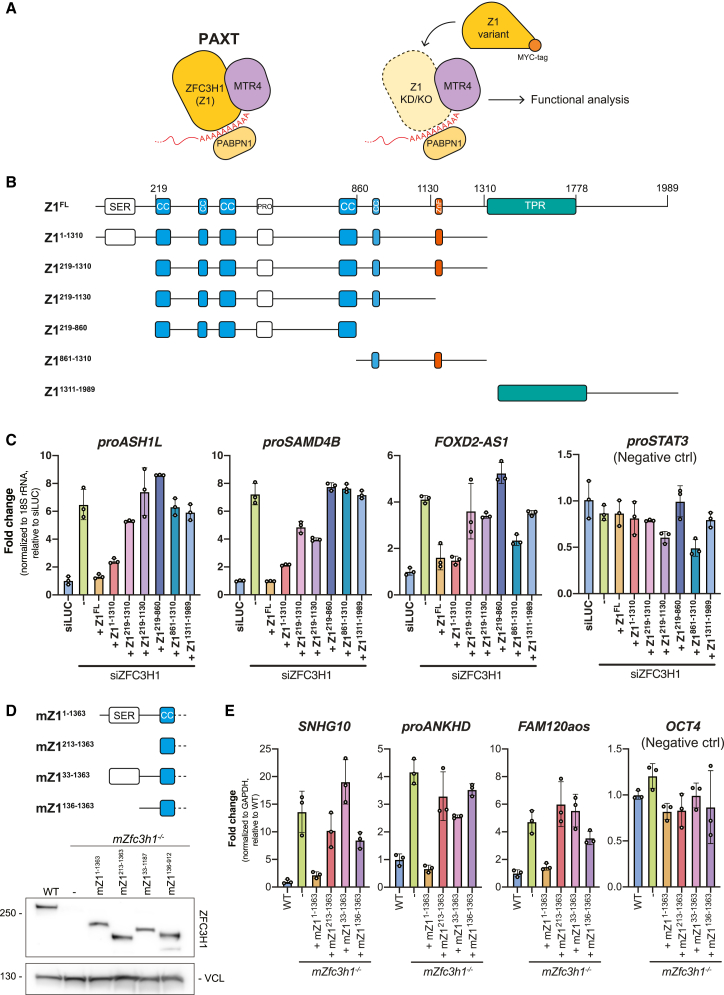
The ZFC3H1 N terminus harbors important information for its function in RNA decay (A) Left: schematic representation of the PAXT connection, highlighting its ZFC3H1-MTR4 core. See text for more detail. Right: experimental design of the ZFC3H1 mutational analysis where the endogenous protein was replaced by one of the MYC-tagged variants from (B). (B) Schematic map of full-length ZFC3H1 (Z1^FL^), depicting its predicted domains: SER, serine-rich; CC, coiled coil; PRO, proline-rich; ZnF, zinc finger; TPR, tetratricopeptide repeat and the regions of ZFC3H1 covered by the generated Z1 variants. (C) qRT-PCR analysis of selected PAXT substrates (proASH1L, proSAMD4B, FOXD2-AS1) and a negative control NEXT substrate (proSTAT3) from total RNA isolated from HeLa cells treated with control (siLUC) or ZFC3H1-targeting siRNA, while stably expressing one of the siRNA-immune ZFC3H1 variants from (B). The RT step was performed using a mixture of random and oligo d(T)_20_VN primers. qPCR amplicons were positioned in TU 5′-end regions. Results were normalized to 18S rRNA levels and plotted as fold change relative to siLUC control samples. Columns represent the mean values of three technical replicates, which are depicted as individual data points, with error bars showing the standard deviation (SD). (D) Top: schematic representation of the mouse Z1^1-1363^ variant and its N-terminal truncations used for further characterization of ZFC3H1. Bottom: western blotting (WB) analysis of expression levels of mouse ZFC3H1 variants in *mZfc3h1*^*−/−*^ mESCs, using antibodies against ZFC3H1 and vinculin (VCL) as a loading control. (E) qRT-PCR analysis of selected mouse PAXT substrates and OCT4 mRNA (negative control) from total RNA isolated from cells used in (D). Results were normalized to GAPDH mRNA levels and plotted as fold changes relative to WT control samples. Data representation as in (C).

Nuclear RNA decay is primarily handled by the 3′-5′ exonucleolytic activity of the RNA exosome complex.^17^^,^^19^ To gain access to its RNAPII-derived substrates, the exosome-associated RNA helicase MTR4^20^^,^^21^^,^^22^ can contact at least two distinct nucleoplasmic adaptors; the nuclear exosome targeting (NEXT) complex^23^ and the poly(A) exosome targeting (PAXT) connection,^24^^,^^25^ targeting pA^−^ and pA^+^ transcripts, respectively.^3^^,^^26^ The NEXT complex, formed by a dimer of MTR4-ZCCHC8-RBM7 heterotrimers,^27^^,^^28^ can be recruited to short, TSS-proximal, pA^−^ transcripts by connecting to the RNA-bound cap-binding complex (CBC) via the ARS2 and ZC3H18 proteins.^3^^,^^29^^,^^30^ The PAXT connection, on the other hand, consists of a core MTR4-ZFC3H1 heterodimer that associates with the nuclear poly(A) binding protein (PABPN1) in an RNA-dependent manner (Figure 1A, left). In addition, less-well-described interactions with the RNA binding proteins ZC3H3, RBM26, or RBM27 are suggested to occur. Together, this facilitates the decay of a wide range of pA^+^ RNAs.^24^^,^^25^^,^^31^^,^^32^

The 5′ end of every RNAPII-produced transcript, regardless of its stability, is m^7^G cap modified and nascently bound by the CBC during the early stages of transcription.^33^ The CBC in turn associates with ARS2, forming the trimeric CBCA complex,^29^^,^^34^^,^^35^ which acts as a central hub for the competitive exchanges of RNA sorting factors, also termed “classifiers,”^36^ that ultimately direct the RNA toward a productive or a destructive fate.^18^ For example, CBCA can connect to factors, such as ALYREF, PHAX, or FLASH, to promote the cellular transport of mRNA, snRNA, or replication-dependent histone (RDH) RNA, respectively.^18^^,^^36^ Alternatively, CBCA can direct its bound transcripts toward RNA decay.^29^ A key factor facilitating this is ZC3H18, which was shown to connect the CBCA and NEXT complexes,^29^^,^^37^ while directly competing with productive CBCA interactors such as PHAX.^38^^,^^39^ While the CBCA-ZC3H18-NEXT association was validated through protein domain mapping,^37^ an analogous connection to PAXT was merely suggested based on the presence of ZC3H18 in PAXT interactome data but has lacked further experimental validation.^24^ Indeed, PAXT activity was originally proposed to rely on prolonged RNA nuclear residence times as a means of purging nuclei of pA^+^ transcripts inefficiently managed by export factors, such as ALYREF.^40^^,^^41^^,^^42^^,^^43^ Hence, bona fide PAXT substrates were considered longer and more processed than their NEXT-sensitive counterparts.^24^ More recent genome-wide analyses have, however, revealed that PAXT also targets short and unspliced RNAs which, apart from their pA^+^ 3′-end status, are biochemically reminiscent of NEXT substrates.^3^^,^^26^ Whether these TSS-proximal pA^+^ transcripts rely on the same recruitment mechanism as longer and processed PAXT substrates, and which role, if any, ZC3H18 plays in the process, remains unclear.

Here, we find that the N-terminal domain of ZFC3H1 is instrumental for PAXT targeting of short pA^+^ transcripts. This is achieved by its direct binding to ARS2 via a conserved acidic-rich short linear motif (SLiM), consistent with observations reported in ZFC3H1 homologs.^44^^,^^45^ Surprisingly, however, we find that ZC3H18, which promotes NEXT-mediated RNA decay, exhibits an inhibitory effect on the ARS2-dependent PAXT decay pathway. This inhibition is explained by a competition between ZFC3H1 and ZC3H18, both of which utilize a similar SLiM to bind a common surface on ARS2. We suggest that this intricate setup provides the possibility for increased NEXT activity to lift the ZC3H18 inhibition of PAXT, thereby providing for co-activation of the two pathways in situations where the demand for RNA turnover is high.

## Results

### The ZFC3H1 N terminus harbors important information for its function in RNA decay

ZFC3H1 is the central hub of PAXT, mediating direct contact with MTR4 and further associating with accessory PAXT components^25^^,^^46^ (Figure 1A, left). To dissect the relative contributions of ZFC3H1 domains in RNA decay, we examined ZFC3H1 through the domain-predicted generation of six C-terminally MYC-tagged ZFC3H1 variants (Z1^x^) (Figure 1B). These truncated proteins, as well as their full-length (FL) counterpart, were individually and stably expressed in HeLa cells depleted of endogenous ZFC3H1 using UTR-specific siRNA (siZFC3H1) (Figure S1A). Possible functional complementation was then assessed by qRT-PCR using primers toward known PAXT substrates (Figure 1A, right), which revealed that only the Z1^FL^ and Z1^1-1310^ variants consistently repressed the upregulation of RNA levels induced by siZFC3H1 (Figure 1C). This activity was particularly significant for the Z1^1-1310^ variant due to its relatively low expression (Figure S1A). In contrast, higher expression of the N-terminally truncated Z1^219-1310^ variant yielded only marginal activity, which immediately highlighted the N terminus (1–218 aa) of ZFC3H1 as being important for targeting of the tested substrates (Figure 1C). Due to the somewhat variable expression levels of the ZFC3H1 variants in HeLa cells (Figure S1A), we validated these results by introducing homologous mouse (mZ1^x^) constructs into our previously established ZFC3H1 knockout mouse embryonic stem cells (mESCs)^47^ (Figure S1B). With a caveat that we could not achieve expression of mZ1^FL^ or mZ1^1364-1992^, we consistently observed that only the mZ1^1-1363^ variant, containing the intact N terminus, was able to repress the upregulation of PAXT targets triggered by the *Zfc3h1*^*−/−*^ conditions (Figure S1C). Using the mESC system, we further narrowed down the functional region by interrogating activities of two additional N-terminal truncations of mZ1^1-1363^ (mZ1^33-1363^ and mZ1^136-1363^, Figure 1D), which revealed that the first 33 aa of ZFC3H1 are important for its function (Figure 1E). From this, we conclude that the extreme N terminus of ZFC3H1 plays an important role in the decay of select PAXT-sensitive RNAs.

### A conserved short linear motif connects ZFC3H1 to the CBC via ARS2

**Figure 2:**
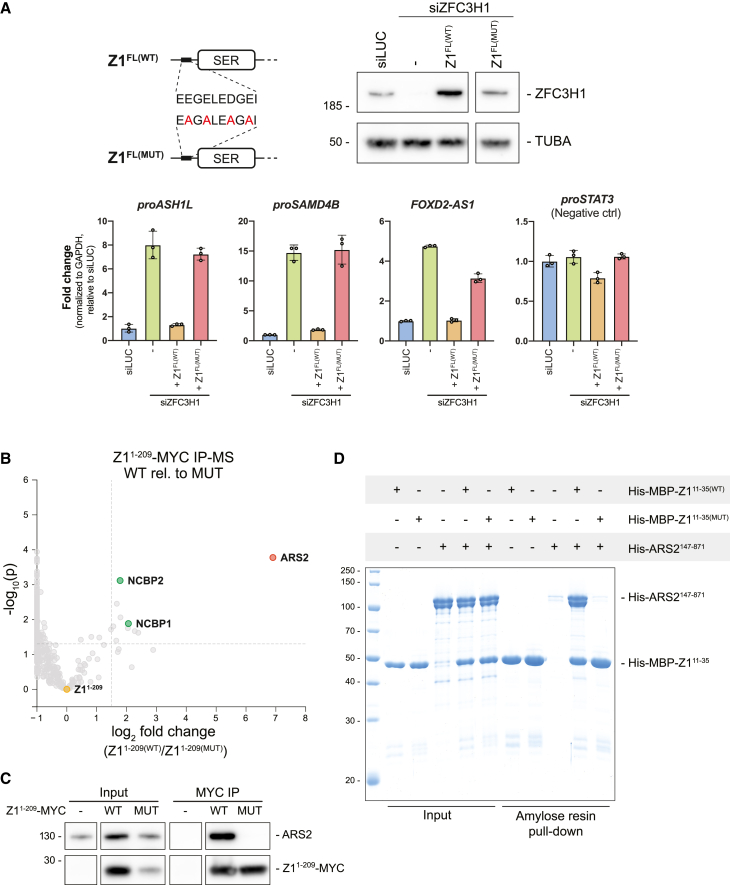
A conserved short linear motif connects ZFC3H1 to the CBC via ARS2 (A) Top left: schematic representation showing the conserved acidic short linear motif (SLiM) in Z1^FL(WT)^ and the point mutations introduced in the Z1^FL(MUT)^ construct. Top right: WB analysis of Z1^FL(WT)^ and Z1^FL(MUT)^ expression in siZFC3H1-treated cells, using antibodies against ZFC3H1 and tubulin alpha (TUBA) as a loading control. siLUC- and siZFC3H1-treated cells served as controls. Irrelevant samples between the “Z1^FL(WT)^” and “Z1^FL(MUT)^” lanes were deleted. Bottom: qRT-PCR analysis as in Figure 1C but on RNA from the above cell lines. Results were normalized to GAPDH mRNA levels. (B) Volcano plot of mass spectrometry (MS) analysis of three biological replicates of MYC IPs from lysates of cells expressing MYC-tagged Z1^1-209(WT)^ relative to Z1^1-209(MUT)^. Protein signals from both samples were normalized to bait protein levels. (C) WB analysis of Z1^1-209(WT)^ and Z1^1-209(MUT)^ MYC-IP samples from (B). The input and IP samples were probed with antibodies against MYC and ARS2. Irrelevant samples between control (−) and “Z1^1-209(WT)^” lanes were deleted. Note that signals from input and IP samples were captured at different exposure times. (D) Coomassie-stained SDS-PAGE gel showing input and amylose resin pull-down samples from assays of His-ARS2^147-871^ incubated with His-MBP-Z1^11-35(WT)^ or His-MBP-Z1^11-35(MUT)^. “+” on top indicates which proteins were added. Protein markers are indicated to the left.

To further investigate the N terminus of ZFC3H1, we first addressed the conservation of this region by multiple sequence alignment analysis. This revealed tandem copies of a highly conserved and acidic SLiM within the first 33 aa of ZFC3H1 (Figure S2A). Interestingly, an identical motif was previously highlighted in the *S. pombe* ZFC3H1 homolog, spRed1, where it was linked to the binding of the spArs2 protein.^44^ Moreover, while our study was under completion, such ARS2 binding was confirmed by *in vitro* assays using human proteins.^45^ Inspired by this, we introduced point mutations into the SLiM of Z1^FL^ (Figure 2A, upper left) and assessed their impact by expressing the mutant protein Z1^FL(MUT)^ in HeLa cells depleted of endogenous ZFC3H1 (Figure 2A, upper right; Figure S2B). Like the N-terminal deletions, the Z1^FL(MUT)^ construct was unable to repress RNA levels of selected PAXT substrates in siZFC3H1-treated cells (Figure 2A, lower).

To interrogate the effect of these point mutations on the interactome of the ZFC3H1 N terminus, we generated wild-type (WT) (Z1^1-209(WT)^) and mutant (Z1^1-209(MUT)^) MYC-tagged variants and subjected them to immunoprecipitation (IP) followed by mass spectrometry (IP-MS). Plotting the relative enrichment of interacting proteins in Z1^1-209(WT)^ vs. Z1^1-209(MUT)^ IPs, we observed a significant enrichment of ARS2 and CBC components, NCBP1 and NCBP2 (Figure 2B). This interaction of ARS2 with the acidic-rich N terminus of ZFC3H1 was confirmed by IP-WB analysis (Figures 2C and S2C) and by *in vitro* pull-downs, demonstrating that the recombinant Z1^11-35(WT)^, but not Z1^11-35(MUT)^, directly interacts with ARS2^147-871^ (Figure 2D). To finally demonstrate that the conserved SLiM of ZFC3H1 is not only sufficient but also necessary for ARS2 binding, we performed GFP-ARS2 IPs from cells expressing either Z1^FL(WT)^ or Z1^FL(MUT)^. As expected, the Z1^FL(MUT)^ protein did not co-immunoprecipitate (coIP) with ARS2 (Figure S2D). Consistent with previous studies, we therefore conclude that the conserved acidic SLiM of ZFC3H1 binds ARS2 directly and further that this interaction is functionally relevant.

### The ZFC3H1-ARS2 connection facilitates the decay of short, adenylated transcripts

**Figure 3:**
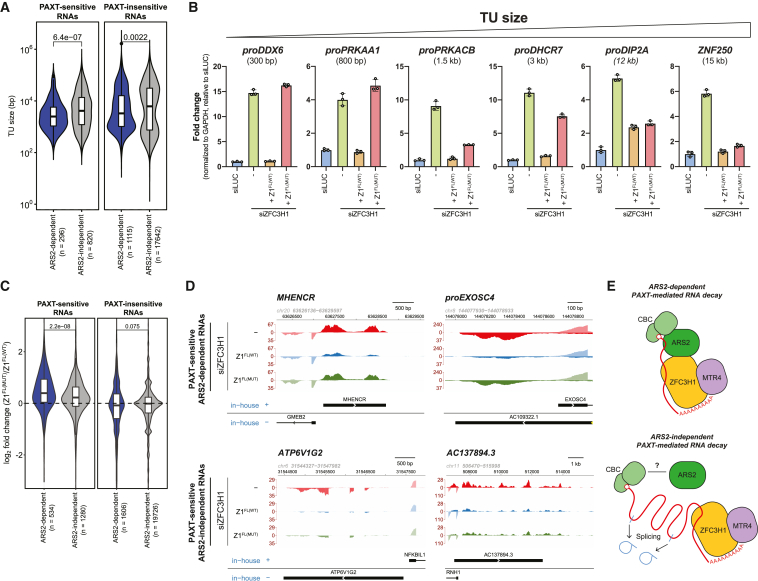
The ZFC3H1-ARS2 connection facilitates the decay of short, adenylated transcripts (A) Violin boxplots depicting transcription unit (TU) size distribution of PAXT-sensitive and -insensitive RNAs^3^ further categorized into ARS2-dependent and -independent groups as indicated. The following criteria were applied to define ARS2-dependent TUs: log_2_ fold change (siARS2/CTRL) > 0 and adjusted p value > 0.1 in total RNA-seq dataset generated from HeLa cells depleted of ARS2.^30^ TUs exhibiting NEXT sensitivity were excluded from both transcript categories. p values, calculated using two-sided Student’s t test and indicating the difference between ARS2-dependent and -independent groups, are displayed on top of the plots. (B) qRT-PCR analysis of selected PAXT substrates derived from TUs of different sizes as estimated from RNA-seq data from siZFC3H1 cells.^24^ RNA samples and data representation as in Figure 2A. qPCR amplicons for proDIP2A and ZNF250 were positioned in TU 3′-end regions to avoid detection of early terminated RNA isoforms. (C) Violin boxplots depicting the log_2_ fold change distributions of ARS2-dependent and -independent PAXT-sensitive and -insensitive RNAs from total RNA-seq datasets generated from siZFC3H1-treated HeLa cells, expressing Z1^FL(MUT)^ relative to Z1^FL(WT)^. TUs exhibiting NEXT sensitivity were included in all categories as NEXT-mediated RNA decay was not disrupted in these samples. p values were calculated as in (A). (D) Genome browser views of representative ARS2-dependent and -independent PAXT-sensitive transcripts from RNA-seq dataset from (C). Tracks show the average signal of three biological replicates in the relevant genomic coordinates. Signals from “+” and “−” strands are displayed as positive and negative values, respectively. The irrelevant strand signal has reduced opacity. An in-house custom HeLa-specific transcriptome annotation^9^ is displayed for both strands below the tracks. (E) Model of PAXT-mediated RNA decay. The ARS2-dependent branch utilizes the ZFC3H1-ARS2 connection to enhance targeting of primarily short, unspliced substrates (top). The ARS2-independent branch targets mainly longer and spliced RNAs (bottom). Whether ARS2 still binds the ARS2-independent substrates is unclear (?).

What might then be the mechanistic consequence of the ZFC3H1-ARS2 interaction, given that ZFC3H1/MTR4 was assumed to gain substrate access via the RNA pA tail and its associated PABPN1^24^? Providing a clue, we recently reported that artificial tethering of ARS2 to a pA^+^ reporter RNA made PAXT-mediated decay more efficient.^48^ Hence, we set out to determine the exact role of ARS2 in PAXT-mediated RNA decay by revisiting our previously published RNA sequencing (RNA-seq) data from siARS2-treated HeLa cells.^30^ Utilizing a set of PAXT-sensitive targets, defined as upregulated upon depletion of ZFC3H1 and ZC3H3,^3^ a total of 1,116 was split into 296 ARS2-dependent and 820 ARS2-independent substrates (Figure 3A). Stratifying these RNAs by their corresponding TU lengths revealed that ARS2-dependent PAXT targets were generally shorter (Figures 3A and S3A, left) and with fewer exons (Figure S3B, left) than their ARS2-independent counterparts. In this way, ARS2 depletion appeared to primarily affect shorter substrates as also seen for the NEXT pathway (Figures S3A and S3B, right). We note that this trend was, to a milder extent, also observed in the control set of PAXT-insensitive RNAs (Figure 3A, right), consistent with the idea that ARS2 impacts the metabolism of a wider variety of short RNAs.

As ARS2 is a central RNA sorting factor,^36^ its depletion will affect several aspects of RNA metabolism. We therefore investigated this size-based substrate trend by analyzing RNA isolated from siZFC3H1 HeLa cells complemented with either FL ZFC3H1 (Z1^FL(WT)^) or its mutated counterpart incapable of binding ARS2 (Z1^FL(MUT)^). As the qRT-PCR amplicons utilized in our earlier analysis of the Z1^FL(MUT)^ construct (Figure 2A) were designed to detect short PAXT substrates, we here included amplicons detecting PAXT substrates of increasing lengths. This strategy revealed that, while the Z1^FL(MUT)^ variant was unable to target short PAXT-sensitive transcripts, it was fully capable of repressing such longer RNAs (Figure 3B). We further analyzed the functionality of the Z1^FL(MUT)^ construct in the decay of ARS2-independent substrates at a global scale by conducting triplicate RNA-seq experimentation of total RNA purified from the Z1^FL(WT)^- and Z1^FL(MUT)^-expressing siZFC3H1-treated cells (Figure S3C). As predicted, ARS2-dependent PAXT substrates were significantly upregulated upon Z1^FL(MUT)^ relative to Z1^FL(WT)^ expression, while a less pronounced effect was observed for ARS2-independent RNAs (Figures 3C and 3D). The latter might, at least partly, result from the lower expression levels of the Z1^FL(MUT)^ protein (Figure 2A, upper right).

Because the CBCA complex could, in principle, suffice for recruitment of ZFC3H1 to ARS2-dependent substrates, we investigated the extent to which PABPN1 would also be required for the decay of these RNAs. To this end, we utilized previous siPABPN1 RNA-seq data,^24^ which revealed that PABPN1 depletion results in the upregulation of both ARS2-dependent and -independent PAXT substrates (Figure S3D).

We conclude that at least two independent branches of PAXT-mediated RNA decay exist. One takes advantage of the CBCA connection to enhance access to shorter substrates, resembling polyadenylated versions of those targeted by the NEXT pathway (Figure 3E, top). The other branch targets longer and more spliced RNAs, possibly relying more on the pA tail for factor recruitment (Figure 3E, bottom).

### ZC3H18 antagonizes the ARS2-dependent PAXT pathway

**Figure 4:**
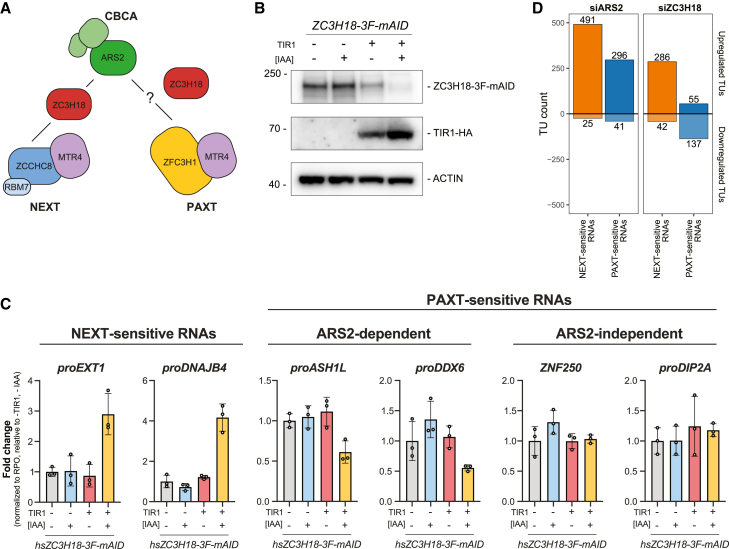
ZC3H18 antagonizes the ARS2-dependent PAXT pathway (A) Schematic representation of the CBCA-NEXT and CBCA-PAXT connections. ZC3H18 bridges the CBCA and NEXT complexes, while its role for CBCA-PAXT was evaluated here. (B) WB analysis showing depletion of ZC3H18-3F-mAID in HeLa cells with (+) or without (−) expression of TIR1-HA and treatment with IAA as indicated. Membranes were probed with antibodies against FLAG, HA, and ACTIN as a loading control. (C) qRT-PCR analysis of selected NEXT-sensitive (left) and ARS2-dependent (middle) or -independent (right) PAXT-sensitive RNAs from total RNA isolated from cells used in (B). Results were normalized to RPO mRNA levels and plotted as fold change relative to -TIR1, -IAA control samples. Data representation as in Figure 1C. (D) Bar plots showing TU counts of NEXT-sensitive (orange) and PAXT-sensitive (blue) RNAs upregulated (>0) or downregulated (<0) in total RNA-seq datasets generated from HeLa cells depleted of ARS2 or ZC3H18 relative to control cells.^30^

The physical link between the CBCA and NEXT complexes was reported to be bridged by the ZC3H18 protein (Figure 4A), the depletion of which results in the accumulation of NEXT substrates.^15^^,^^29^^,^^37^ A comparable connection between ZC3H18 and ZFC3H1 was also speculated to link the CBCA to PAXT^24^; however, this idea was never further elaborated. Given these biochemical interactions and our finding that NEXT- and ARS2-dependent PAXT substrates are of largely similar lengths, we were prompted to investigate how ZC3H18 might impact ARS2-dependent PAXT activity.

Our previous approach to study ZC3H18 function utilized siRNA-mediated factor depletion, which led to significant, albeit somewhat moderate increases of NEXT-sensitive RNAs in siZC3H18 conditions.^29^^,^^30^^,^^37^ We therefore turned to a conditional degron-based technique to rapidly deplete endogenous ZC3H18 via a fused mini-auxin-inducible degron and 3×FLAG (3F-mAID) tag.^49^ After 16 h of auxin (IAA) treatment, ZC3H18-3F-mAID protein levels were efficiently depleted (Figure 4B). Subsequent qRT-PCR assessment of selected NEXT-sensitive RNAs revealed their significant, and expected, upregulation (Figure 4C, left). In contrast, and to our surprise, levels of ARS2-dependent PAXT substrates were reduced upon ZC3H18 depletion (Figure 4C, middle). Thus, ZC3H18 may normally antagonize decay of these transcripts. Because levels of ARS2-independent PAXT substrates yielded no significant effect upon ZC3H18 depletion (Figure 4C, right), the inhibitory function of ZC3H18 might be enacted via ARS2. To validate these findings, we generated analogous *Zc3h18-3F-mAID* mESCs and included previously established *Zcchc8-3F-mAID* and *Zfc3h1-3F-mAID* lines^50^ to perform a comparative rapid factor depletion time course (Figure S4A). Consistent with our HeLa cell data, NEXT-sensitive RNAs (FigureS4B, top: *proNUP85*, *proSMG5*) were increasingly upregulated over the 8-h time course following depletion of ZC3H18 and ZCCHC8, while PAXT (ZFC3H1)-sensitive RNAs (Figure S4B, bottom: *FAM120aos*, *proANKHD1*) were reduced by ZC3H18 depletion.

To corroborate these diverse functions of ZC3H18 in NEXT- and PAXT-mediated RNA decay genome-wide, we revisited siZC3H18 RNA-seq data^30^ and assessed changes in NEXT- or PAXT-sensitive transcripts in siARS2 vs. siZC3H18 conditions. In general, NEXT-sensitive RNAs were upregulated in both siARS2 and siZC3H18 conditions (Figure 4D, orange bar plots). PAXT-sensitive RNAs, on the other hand, were only preferentially upregulated in siARS2 conditions but predominantly downregulated upon ZC3H18 depletion (Figure 4D, blue bar plots). Thus, while ZC3H18 promotes NEXT activity it rather appears to inhibit the ARS2-dependent PAXT pathway.

### ZFC3H1 and ZC3H18 compete for ARS2 binding

**Figure 5:**
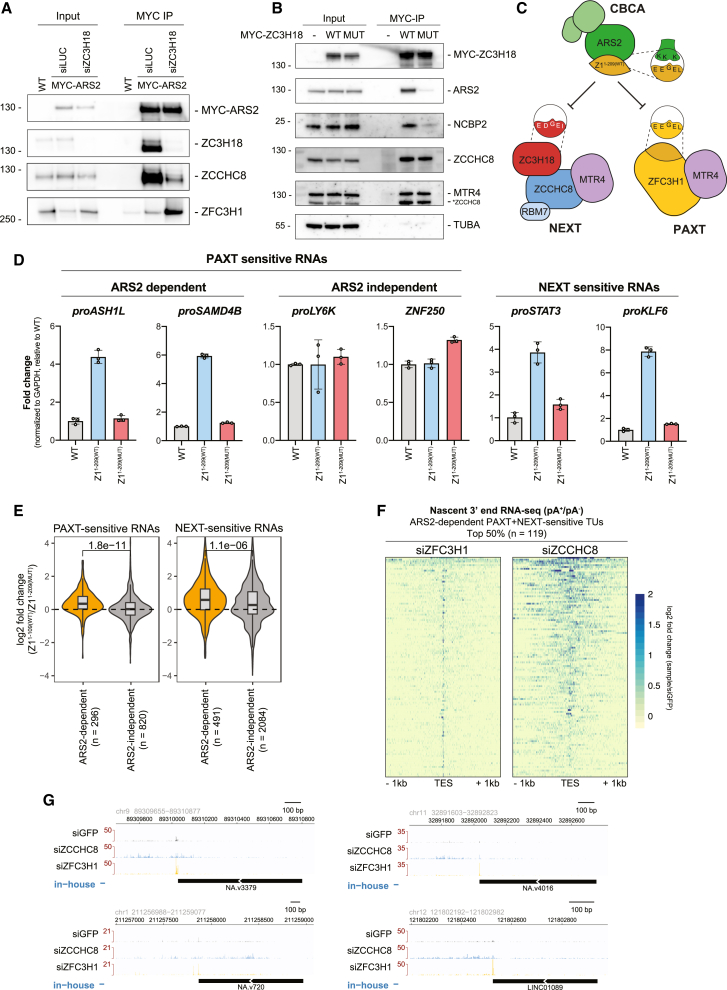
ZFC3H1 and ZC3H18 compete for ARS2 binding (A) WB analysis of MYC IPs from lysates of HeLa cells stably expressing MYC-ARS2 and treated with either control siRNA (siLUC) or siZC3H18. Input and IP samples were probed with antibodies against MYC, ZC3H18, ZCCHC8, and ZFC3H1 as indicated. (B) WB analysis of MYC IPs from lysates of HeLa cells expressing MYC-tagged ZC3H18^WT^, MYC-tagged ZC3H18^MUT^, and untagged control cells. Input and IP samples were probed with antibodies against MYC, ARS2, NCBP2, ZCCHC8, MTR4, and tubulin alpha (TUBA) as a control. The membrane probed for ZCCHC8 was incompletely stripped and subsequently probed for MTR4 resulting in detection of residual ZCCHC8 signal as indicated. (C) Schematic representation of the suggested inhibition of a common ZFC3H1/ZC3H18 binding site on ARS2 by overexpression of Z1^1-209(WT)^. The zoom-ins depict the conserved ARS2-binding SLiM in the indicated proteins and the corresponding binding site on ARS2. (D) qRT-PCR analysis as in Figure 4E but of total RNA isolated from WT HeLa control cells or from cells following 2 days of overexpression of stably integrated Z1^1-209(WT)^ or Z1^1-209(MUT)^. (E) Violin boxplots depicting log_2_ fold change distribution of PAXT- (left) and NEXT-sensitive (right) RNAs in a total RNA-seq dataset generated from cells used in (D). Both RNA groups were further categorized into ARS2 dependent and independent as in Figure 3A. p values, calculated as in Figure 3A, indicate the difference between ARS2-dependent and -independent groups. (F) Heatmaps displaying log_2_ fold changes of 3′ end 4-thiouridine RNA-seq data from siZFC3H1 (left) and siZCCHC8 (right) samples relative to their control.^3^ Data were plotted in 2-kb regions centered around annotated TESs of the top 50% of ARS2-dependent PAXT- and NEXT-sensitive TUs. The average signals from three replicates are shown. RNA samples were *in vitro* polyadenylated to capture both pA^+^ and pA^−^ transcripts. (G) Genome browser views of representative TUs from (F). Tracks contain the average signal of three biological replicate samples showing the relevant strand and genomic coordinates. The in-house annotation from Figure 3D is displayed for the relevant strand below the tracks.

As ZC3H18 only antagonizes the ARS2-dependent branch of the PAXT pathway, we tested whether ZC3H18 might negatively affect ARS2-ZFC3H1 association by performing IPs of MYC-ARS2 stably integrated in HeLa following control of ZC3H18 levels by siRNA depletion. Consistent with its role in bridging the CBCA and NEXT complexes, ZC3H18 depletion led to reduced ARS2-ZCCHC8 association (Figures 5A and S5A). Conversely, levels of ZFC3H1 were increased in ARS2 IPs when ZC3H18 was depleted. The observed downregulation of PAXT-sensitive RNAs in this condition (Figure 4C) was therefore likely to be due to the reinforced interaction between ARS2 and ZFC3H1, which in turn enhances ARS2-dependent PAXT activity.

In line with the possibility that ZC3H18 and ZFC3H1 might compete for binding to the same region of ARS2, sequence analysis of ZC3H18 revealed three copies of a ZFC3H1-like acidic SLiM in a highly conserved region of the protein (Figure S5B). To examine whether this motif indeed contributes to the ZC3H18-ARS2 interaction, we introduced diagnostic point mutations into this putative ZC3H18 acidic SLiM (ZC3H18^MUT^) analogous to the mutations of Z1^FL(MUT)^ (Figures 2A and S5B). We then stably integrated MYC-tagged ZC3H18^WT^ and ZC3H18^MUT^ variants into HeLa cells and performed MYC IPs. Both ZC3H18^WT^ and ZC3H18^MUT^ constructs were expressed at comparable levels and co-immunoprecipitated ZCCHC8 and MTR4 with similar efficiencies (Figure 5B), demonstrating that the introduced changes did not affect NEXT binding. However, in contrast, ARS2 and NCBP2 levels were both significantly reduced in the ZC3H18^MUT^ IP, confirming that ZC3H18 and ZFC3H1 utilize similar motifs to interact with ARS2. Moreover, like the ZFC3H1 acidic SLiM, the ZC3H18 SLiM was also sufficient to bind ARS2 *in vitro* in a WT-specific manner (Figure S5C^15^). We note that a previous study reported a coiled-coil ZC3H18 domain outside of this conserved region to contribute to ARS2 binding,^37^ the relevance of which now remains to be further investigated. Finally, we performed *in vitro* pull-downs of WT SLiM containing ZFC3H1 and ZC3H18 peptides in combination with either a WT ARS2^147-871^ peptide or a ZnF domain mutant (K719A, K722A, K734A), previously shown to disrupt binding between ARS2 and effector proteins.^15^^,^^45^^,^^48^^,^^51^ Here, we reassuringly confirmed that ZFC3H1 and ZC3H18 SLiM peptides both bound to WT ARS2, while these interactions were lost with the ZnF mutant (Figure S5D). We conclude that both ZC3H18 and ZFC3H1 can directly interact with the ZnF/effector domain of ARS2 via their acidic SLiM motifs.

Given the competition between ZFC3H1 and ZC3H18 for ARS2 binding, one might expect that decreased ZFC3H1 levels would consequently affect NEXT activity. However, ZFC3H1 depletion did not significantly affect NEXT-sensitive transcripts,^3^^,^^24^ possibly because ZFC3H1 protein is only approximately half as abundant as ZC3H18 in HeLa cells.^52^ We therefore turned to overexpression of the Z1^1-209(WT)^ variant containing the ARS2-binding motif. As Z1^1-209(WT)^ cannot form the full PAXT connection, we assumed that its overexpression might inhibit both NEXT- and PAXT-mediated RNA decay pathways by saturating the corresponding binding site on ARS2 (Figure 5C). Hence, we performed MYC-ARS2 IPs following DOX-induced overexpression of the Z1^1-209(WT)^ variant, which significantly reduced the association of ZC3H18 and by extension ZCCHC8 with ARS2 (Figure S5E). Furthermore, this also reduced the association of endogenous ZFC3H1 with MYC-ARS2, thereby reducing both NEXT and PAXT proteins on ARS2. To validate the functional consequences of this, we analyzed RNA from equivalent samples while also utilizing the Z1^1-209(MUT)^, deficient for ARS2 binding (Figures 2C and 2D), as a negative control. As expected, both NEXT-sensitive and ARS2-dependent PAXT-sensitive RNAs were upregulated upon overexpression of Z1^1-209(WT)^, while Z1^1-209(MUT)^ overexpression showed no significant substrate changes (Figure 5D). In addition, ARS2-independent PAXT substrates remained unaffected, supporting the idea that the observed phenotype was due to the blocking of the common ZFC3H1/ZC3H18 interaction surface on ARS2. To obtain a global view, we further performed RNA-seq analysis of the same samples (Figure S3C), which confirmed a Z1^1-209(WT)^ overexpression-specific upregulation of both NEXT- and PAXT-sensitive ARS2-dependent RNAs, while no significant changes were observed for ARS2-independent substrates (Figure 5E).

At first glance, it seemed counterintuitive for two RNA decay pathways to compete for ARS2-bound RNAs. However, because PAXT and NEXT target distinct substrate species (pA^+^ vs. pA^−^ RNAs, respectively), this could possibly be rationalized by individual TUs expressing transcripts sensitive to both pathways.^3^ To provide a visual demonstration of this phenomenon, we stratified TUs sensitive to both PAXT and NEXT, adding a further criteria of ARS2 sensitivity (n = 238). Transcript production from this TU selection was then analyzed using our previously published 3′-end 4-thiouridine RNA-seq data from HeLa cells depleted of ZFC3H1 or ZCCHC8.^3^ To capture both non-adenylated and adenylated transcript 3′ ends (pA^−/+^), we utilized libraries generated from RNAs following their *in vitro* pA.^53^ 3′-end RNA-seq signals from siZFC3H1 and siZCCHC8 relative to control samples falling within a 2-kb region surrounding annotated transcript end sites (TESs) were then defined, and the top 50% ARS2-dependent PAXT- and NEXT-sensitive TUs were visualized (Figure 5F). This representation, as well as selected genome browser views (Figure 5G), highlighted the ample presence of dual PAXT- and NEXT-sensitive TUs, which were largely driven by ZFC3H1-sensitive 3′ ends at annotated TESs embedded in more heterogeneous ZCCHC8-sensitive 3′ ends.

**Figure 6:**
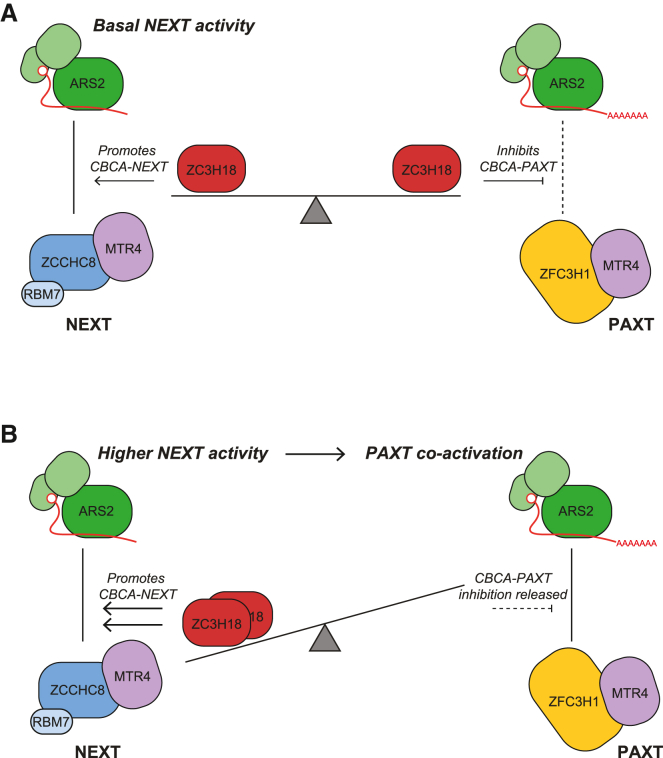
Dual agonistic and antagonistic roles of ZC3H18 provides for co-activation of NEXT and PAXT pathways (A) At TUs with basal NEXT activity ZC3H18 is in excess, resulting in promotion of CBCA-NEXT formation, while inhibiting the CBCA-PAXT connection. (B) Higher substrate load demands increased NEXT activity, which will titrate ZC3H18 from its CBCA-PAXT inhibition and lead to co-activation of the ARS2-dependent PAXT- and NEXT-mediated pathways.

Having ZC3H18 inhibit the ARS2-dependent PAXT pathway would be detrimental for the efficient removal of transcripts produced from such dually sensitive loci. We therefore suggest that demanding engagement of ZC3H18 with NEXT will locally prevent its PAXT-inhibitory function, allowing for co-activation of the PAXT-ARS2 pathway to degrade pA^+^ transcripts generated from the same TU (Figure 6, see discussion).

## Discussion

The PAXT connection targets a variety of nuclear pA^+^ RNAs. At one end, competition between PAXT and nuclear export factors^42^^,^^43^^,^^54^ has led to a proposed nuclear timer model in which PAXT assembles on pA^+^ RNA with a prolonged nuclear residence time due to inefficient export.^18^^,^^40^^,^^41^ Now, we show that PAXT targeting can be enhanced by binding of the core PAXT protein ZFC3H1 to the CBC-associated protein ARS2, providing a more direct response to the production of early terminated and short pA^+^ RNAs. Remarkably, this distinct branch of the PAXT pathway is antagonized by the NEXT-associated ZC3H18 protein via an unprecedented crosstalk between the two RNA decay factors ZFC3H1 and ZC3H18 on CBCA-bound RNAs. While the opposing roles of ZC3H18 in PAXT- and NEXT-mediated RNA decay may at first appear contradictory, we propose that it provides for possible co-activation of both pathways in demanding situations with larger volumes of early terminated RNAs.

Although previous studies of PAXT-mediated RNA decay have acknowledged the existence of a link between CBCA and PAXT, its importance was not subjected to critical scrutiny due to a primary focus on PAXT recruitment via the RNA pA^+^ tail.^24^^,^^25^^,^^46^ However, an artificial PAXT-sensitive RNA reporter system showed a clear requirement for ARS2, implying a role of CBCA in PAXT-mediated RNA decay, at least in some situations.^48^ Moreover, the *S. pombe* CBCA homolog was reported to form a submodule of the Mtl1-Red1 core complex, homologous to the MTR4-ZFC3H1 core of the PAXT connection,^55^ linked via an interaction between spArs2 and spRed1.^44^^,^^45^ Indeed, we confirm here that a group of PAXT-sensitive RNAs display a dependency on ARS2 for their degradation. While we find no categorical difference in biochemical features between ARS2-dependent and -independent PAXT substrates, the former tends to be shorter, less spliced, and more “nascent-like.” This is fitting with the concept of ARS2 being crucial for fate decisions of RNAs produced within a promoter-proximal range of a few kilobases,^15^^,^^30^ which is exemplified by its role in the biogenesis of short functional snRNAs and RDH RNAs,^29^^,^^30^^,^^34^^,^^56^^,^^57^ the termination of short, unstable RNAs,^15^^,^^30^ and the decay of unstable promoter-proximal transcripts via NEXT^3^^,^^15^^,^^24^^,^^30^ or PAXT.^3^ Any role of ARS2 in the metabolism of longer and more processed transcripts remains elusive. We therefore suggest that these depend on features/factors that become more influential once an RNA “moves out” of the ARS2-dependent phase and becomes more of a subject to post-transcriptional processing steps and the assembly into export-competent RNPs. Non-optimal transcripts in this category that fail in any of these productive steps may still fall prey to PAXT-mediated decay^18^ but are not dependent on ARS2 (Figure 3E).

As a biochemical account of the ability of ARS2 to be involved in promotor-proximal RNA metabolism, the CBCA provides a central hub capable of competitive exchanges of multiple RNA sorting factors. Binary competition between ARS2 interactors has been widely reported such as ZC3H18 and PHAX,^38^ ZC3H18 and NCBP3,^39^ and PHAX and NCBP3.^51^ The basis for this competition was shown to be mediated by a common binding surface on ARS2, the lysine-rich surface of the ZnF domain,^15^^,^^45^^,^^48^^,^^51^ which we coin as the “effector” domain. In agreement with recent studies,^44^^,^^45^ we demonstrate here that a highly conserved acidic-rich SLiM in ZFC3H1 is responsible for the direct binding of ARS2. Refined searches of this SLiM consensus ([DE]-[DE]-G-[DE]-[ILV]) have revealed its presence in several other ARS2-interacting proteins involved in nuclear RNA metabolism pathways^15^^,^^44^^,^^45^ and the introduction of diagnostic mutations disrupts their interactions with ARS2. We therefore coin this SLiM motif consensus as an “ARS2 recruitment motif” or ARM. Together, this implies that ZFC3H1 impacts early CBCA-mediated nuclear RNA fate decisions via competition for ARS2 binding with other nuclear RNA sorting factors. Previous data support the notion that these factors do not show preference for specific RNA biotypes during nascent stages of transcription but rather that they dynamically sample and exchange on the CBCA until additional cues are sensed to form a signature that ultimately settles the fate of the transcript.^18^^,^^38^ The close proximity of a pA^+^ tail to the CBCA, along with the association of PABPN1, may be such a cue to tip the scales of competition in favor of the CBCA-PAXT connection, explaining why ARS2-dependent PAXT substrates tend to be short. On the other hand, the presence of introns might result in a preferential recruitment of productive factors to the nascent RNP, thus leaning spliced PAXT substrates toward the ARS2-independent group.

While factor exchange on the ARS2 effector domain was considered a productive vs. destructive RNP concept, we report here the surprising observation that two ARS2-interacting factors, ZFC3H1 and ZC3H18, targeting transcripts for PAXT- and NEXT-mediated degradation, respectively, also compete between themselves. Moreover, we demonstrate that this competition has unprecedented functional consequences for nuclear RNA decay. Based on our findings, we propose a model where basal NEXT activity, under standard conditions, leaves nuclear amounts of ZC3H18 and NEXT components in excess. This, in turn, allows for ZC3H18 molecules, unoccupied by NEXT, to dampen the CBCA-PAXT connection via competitive ARS2 binding (Figure 6A), which is likely possible due to the relatively low nuclear levels of ZFC3H1 compared with ZC3H18 and ZCCHC8.^52^ This inhibition may then be lifted in physiological conditions, or in certain sub-compartments of the nucleus, that demand intense NEXT activity, for example, at loci, yielding a higher load of early terminated transcripts (Figure 6B). Because a large number of TUs produce both NEXT- and PAXT-sensitive transcripts,^3^ an increased production of NEXT substrates is naturally predicted to coincide with a parallel increase in short PAXT-sensitive RNAs.

Our appreciation of the richness of RNA transactions taking place during the early phases of RNAPII transcription has grown dramatically in recent years, and we are now beginning to understand the biochemical underpinnings of the competition/crosstalk occurring between differently involved pathways. While there are most certainly additional biochemical connections to be delineated, future efforts will also start to address how these are orchestrated inside the cell nucleus to achieve sufficient processing and transport of the needed short RNA biotypes while preventing spurious transcripts to overwhelm such production.

### Limitations of the study

Some aspects of this study were based on siRNA-mediated depletion of factors in human cells over 2–3 days. These long-term depletion experiments could be associated with both secondary and compensatory effects. To remedy this, we have complemented our analyses, where possible, using rapid depletion systems or providing orthogonal data in mESCs to support our conclusions.

As ARS2 interacts with a multitude of RNA sorting factors in a mutually exclusive manner, any perturbations of acidic SLiM/ARM-containing proteins through depletion/overexpression may disrupt the balance of ARS2 complexes. In this study, we only address the impact on ARS2-dependent RNA decay via ZFC3H1 and ZC3H18 through their perturbations. Any effects on reported ARS2-dependent RNA processing^34^^,^^57^ or transcription termination activities^15^^,^^16^ were outside the scope of this manuscript.

## STAR★Methods

### Key resources table

**Table:** 

REAGENT or RESOURCE	SOURCE	IDENTIFIER
**Antibodies**		

Mouse monoclonal anti-ACTIN	Sigma-Aldrich	Cat# A2228; RRID: RRID:AB_476697
Mouse monoclonal anti-FLAG M2	Sigma-Aldrich	Cat# F1804: RRID:AB_262044
Rat monoclonal anti-HA	Sigma-Aldrich	Cat# 11867423001; RRID:AB_390918
Rabbit polyclonal anti-MTR4 (SKIV2L2)	Abcam	Cat# ab70551; RRID:AB_1270701
Rabbit monoclonal anti-MYC	Cell Signaling	Cat# 2278; RRID:AB_490778
Rabbit polyclonal anti-ALPHA-TUBULIN	Rockland	Cat# 600-401-880; RRID:AB_2612816
Mouse monoclonal anti-VINCULIN (VCL)	Sigma-Aldrich	Cat# V9131; RRID:AB_477629
Rabbit polyclonal anti-ZC3H18	Sigma-Aldrich	Cat# HPA040847; RRID:AB_10794865
Mouse polyclonal anti-ZCCHC8	Abcam	Cat# ab68739; RRID:AB_1271512
Rabbit polyclonal anti-ZCCHC8	Bethyl	Cat# A301-806A; RRID:AB_1233063
Rabbit polyclonal anti-ZCCHC8	Novus Biologicals	Cat# NB10094995; RRID: AB_1262274
Rabbit polyclonal anti-ZFC3H1	Sigma-Aldrich	Cat# HPA007151; RRID:AB_1846133
Rabbit polyclonal anti-ARS2	GeneTex	Cat# GTX119872; RRID: AB_10720168
Mouse monoclonal anti-GFP (B-2)	Santa Cruz	Cat# sc-9996; RRID:AB_627695
Goat polyclonal anti-rabbit immunoglobulins/HRP	Agilent	Cat# P0448; RRID:AB_2617138
Goat polyclonal anti-mouse immunoglobulins/HRP	Agilent	Cat# P0447; RRID:AB_2617137
Rabbit polyclonal anti-rat immunoglobulins/HRP	Agilent	Cat# P0450; RRID:AB_2630354

**Bacterial and virus strains**		

DH5-alpha competent *E.coli* strain	N/A	N/A
BL21 Star (DE3) pRARE *E.coli* strain	EMBL Heidelberg Core Facility	N/A

**Chemicals, peptides, and recombinant proteins**		

GSK3 inhibitor (CHIR99021)	Sigma-Aldrich	Cat# SML1046
MEK1/2 inhibitor (PD0325901)	Sigma-Aldrich	Cat# PZ0162
Indole-3-acetic acid sodium salt (IAA)	Sigma-Aldrich	Cat# I5148-10G
TRIzol	Thermo Fisher	Cat# 15596026

**Critical commercial assays**		

siLentFect	BioRad	Cat# 1703362
Lipofectamine 3000 Transfection Reagent	Thermo Fisher	Cat# L300001
Viafect Transfection Reagent	Promega	Cat# E4981
TURBO DNase kit	Thermo Fisher	Cat# AM2238
SuperScript III Reverse Transcriptase	Thermo Fisher	Cat# 1808044
Platinum SYBR Green qPCR SuperMix	Thermo Fisher	Cat# 11733046
Benzonase nuclease	Millipore	Cat# 70746
Benzonase nuclease	Merck	Cat# 71206
Bioanalyzer RNA 6000 Nano Kit	Agilent	Cat# NC1783726
Ribolock RNase Inhibitor	Thermo Fisher	Cat# EO0381
cOmplete, Mini, EDTA-free Protease Inhibitor Cocktail	Roche	Cat# 04693159001
NEBuilder HiFi DNA Assembly cloning kit	NEB	Cat# E5520S
GeneJET PCR Purification Kit	Thermo Fisher	Cat# K0701
Purelink Genomic DNA mini purification Kit	Thermo Fisher	Cat# K182001
SuperSignal West Femto Maximum Sensitivity Substrate	Thermo Fisher	Cat# 34096
Protein G Dynabeads	Thermo Fisher	Cat# 10009D
Pierce Anti-*c*-Myc Magnetic Beads	Thermo Fisher	Cat #88842
ChromoTek GFP-Trap Magnetic Particles M-270	Proteintech	Cat #gtdk
NuPAGE LDS Sample Buffer (4x)	Thermo Fisher	Cat# NP0007
NuPAGE Sample Reducing Agent (10x)	Thermo Fisher	Cat# NP0004
Amylose Resin	NEB	Cat# E8021
Superdex 75 Increase 10/300 GL	Cytiva	Cat# 29148721
HiTrap Heparin HP	Cytiva	Cat# 17040601
HIS-Select Nickel Affinity Gel	Millipore	Cat# P6611
MagReSyn HILIC beads	Resyn Biosciences	Cat# MR-HLC002

**Deposited data**		

RNA-seq data	This study	GEO: GSE212557
IP-MS data	This study	PRIDE: PXD045842

**Experimental models: Cell lines**		

HeLa Kyoto	Anthony A. Hyman	N/A
HeLa Kyoto ZFC3H1^FL(WT)^-MYC	This study	N/A
HeLa Kyoto ZFC3H1^FL(MUT)^-MYC	This study	N/A
HeLa Kyoto ZFC3H1^1−1310^-MYC	This study	N/A
HeLa Kyoto ZFC3H1^219−1310^-MYC	This study	N/A
HeLa Kyoto ZFC3H1^219−1130^-MYC	This study	N/A
HeLa Kyoto ZFC3H1^219−860^-MYC	This study	N/A
HeLa Kyoto ZFC3H1^861−1310^-MYC	This study	N/A
HeLa Kyoto ZFC3H1^1311−1989^-MYC	This study	N/A
HeLa Kyoto ZFC3H1^1−209(WT)^-MYC	This study	N/A
HeLa Kyoto ZFC3H1^1−209(MUT)^-MYC	This study	N/A
HeLa Kyoto MYC-ZC3H18^WT^	This study	N/A
HeLa Kyoto MYC-ZC3H18^MUT^	This study	N/A
HeLa Kyoto MYC-ARS2	This study	N/A
HeLa Kyoto ZC3H18-3F-mAID	This study	N/A
HeLa Kyoto ZC3H18-3F-mAID OsTIR1-HA	This study	N/A
Mouse ES-E14TG2A	ATCC	Cat# CRL-1821; RRID:CVCL_9108
Mouse ES-E14TG2A *Zfc3h1−/−* #1	Garland et al.^47^	N/A
Mouse ES-E14TG2A *Zfc3h1−/− MYC-mZ1*^*1−*^*^1363^*	This study	N/A
Mouse ES-E14TG2A *Zfc3h1−/− MYC-mZ1*^*213−*^*^1363^*	This study	N/A
Mouse ES-E14TG2A *Zfc3h1−/− MYC-mZ1*^*213−*^*^1187^*	This study	N/A
Mouse ES-E14TG2A *Zfc3h1−/− MYC-mZ1*^*213−*^*^612^*	This study	N/A
Mouse ES-E14TG2A *Zfc3h1−/− MYC-mZ1*^*913−*^*^1363^*	This study	N/A
Mouse ES-E14TG2A *Zfc3h1−/− MYC-mZ1*^*33−*^*^1363^*	This study	N/A
Mouse ES-E14TG2A *Zfc3h1−/− MYC-mZ1*^*136−*^*^1363^*	This study	N/A
Mouse ES-E14TG2A *OsTIR1-HA*	Garland et al.^50^	N/A
Mouse ES-E14TG2A *OsTIR1-HA Zcchc8-3F-mAID*	Garland et al.^50^	N/A
Mouse ES-E14TG2A *OsTIR1-HA Zfc3h1-3F-mAID*	Garland et al.^50^	N/A
Mouse ES-E14TG2A *OsTIR1-HA Zc3h18-3F-mAID*	This study	N/A

**Oligonucleotides**		

siRNAs oligonucleotides	See Table S1	N/A
sgRNA oligonucleotides	See Table S2	N/A
qRT-PCR oligonucleotides	See Table S3	N/A

**Recombinant DNA**		

pB/TO[ZFC3H1^FL(WT)^-MYC] HYG	This study	N/A
pB/TO[ZFC3H11^FL(MUT)^-MYC] HYG	This study	N/A
pB/TO[ZFC3H1^1−1310^-MYC] HYG	This study	N/A
pB/TO[ZFC3H1^219−1310^-MYC] HYG	This study	N/A
pB/TO[ZFC3H1^219−1130^-MYC] HYG	This study	N/A
pB/TO[ZFC3H1^219−860^-MYC] HYG	This study	N/A
pB/TO[ZFC3H1^861−1310^-MYC] HYG	This study	N/A
pB/TO[ZFC3H1^1311−1989^-MYC] HYG	This study	N/A
pB/TO[ZFC3H1^1−209(WT)^-MYC] HYG	This study	N/A
pB/TO[ZFC3H1^1−209(MUT)^-MYC] HYG	This study	N/A
pB/TO[MYC-ZC3H18^(WT)^] HYG	This study	N/A
pB/TO[MYC-ZC3H18^(MUT)^] HYG	This study	N/A
pB[MYC-mZFC3H1^1−1363^] BLAST	This study	N/A
pB[MYC-mZFC3H1^213−1363^] BLAST	This study	N/A
pB[MYC-mZFC3H1^213−1187^] BLAST	This study	N/A
pB[MYC-mZFC3H1^213−612^] BLAST	This study	N/A
pB[MYC-mZFC3H1^913−1363^] BLAST	This study	N/A
pB[MYC-mZFC3H1^33−1363^] BLAST	This study	N/A
pB[MYC-mZFC3H1^136−1363^] BLAST	This study	N/A
pB[OsTIR1-HA] ZEOCIN	Garland et al.^50^	N/A
pGCT[hZC3H18-3F-mAID] HYG	This study	N/A
pGCT[hZC3H18-3F-mAID] *NEO*	This study	N/A
pGCT[mZC3H18-3F-mAID] HYG	This study	N/A
pGCT[mZC3H18-3F-mAID] *NEO*	This study	N/A
pB[MYC-ARS2] BLAST	This study	N/A
pCDNA5-GFP-ARS2	Melko et al.^48^	N/A
pcDNA5-ZFC3H1-3F	Meola et al.^24^	N/A

**Software and algorithms**		

R	N/A	N/A
RStudio	N/A	N/A
CHOPCHOP (v3)	Labun et al.^58^	https://chopchop.cbu.uib.no/
HISAT2 (v2.2.1)	Kim et al.^59^	https://github.com/DaehwanKimLab/hisat2
DEseq2 (v1.32.0)	Love et al.^60^	https://bioconductor.org/packages/release/bioc/html/DESeq2
seqNdisplayR	N/A	https://github.com/THJlab/seqNdisplayR/
ImageJ (v1.51)	Schneider, Rasband, and Eliceiri^61^	https://ImageJ.nih.gov/ij/
AriaMx (v1.71)	Agilent	https://www.agilent.com/
Graphpad Prism (9.0.0)	Graphpad	https://www.graphpad.com/scientific-software/prism/
Aminode	Chang et al.^62^	http://www.aminode.org/search

### Resource availability

#### Lead contact

Further information and requests for resources and reagents should be directed to and will be fulfilled by the lead contact, Torben Heick Jensen (thj@mbg.au.dk).

#### Materials availability

Resources and reagents used or generated in this study will be available upon request to the lead contact.

### Experimental model and subject participant details

#### HeLa cell culture and transfections

HeLa Kyoto cells and descended cell lines were cultured in DMEM, GlutaMAX (Gibco) supplemented with 1× Pen-Strep (Gibco) and 10% FBS (Gibco) at 37°C, 5% CO_2_. Cells were passaged every 3–4 days by aspirating medium, dissociating cells with 0.05% Trypsin-EDTA (Gibco) briefly at 37°C, resuspending in culture medium and plating an appropriate amount of cell suspension. Cell lines were seeded in 6-well plates with culture medium without Pen-Strep prior to transfection with plasmid DNA using Lipofectamine 3000 (Invitrogen) or with 20 nm siRNA using siLentFect (Bio-Rad) and RPMI 1640 medium (Gibco), according to the manufacturers’ instructions. A list of siRNAs used in this study are included in Table S1. Cells were incubated for 72 h following siRNA transfection to achieve target protein depletions. For antibiotic selection, Hygromycin B (Invitrogen) was used at 200 μg/mL, Zeocin (Invitrogen) was used at 100 μg/mL. For mAID-tagged cell lines, 750 μM Indole-3-acetic acid sodium salt (IAA, Sigma) was supplemented to the medium and cells were incubated for the indicated timepoints before harvest. For transient expression of ZFC3H1 and ARS2, pcDNA5-ZFC3H1-3×FLAG^24^ and pcDNA5-GFP-ARS2^48^ plasmids were transfected using Lipofectamine 3000 (Invitrogen), according to the manufacturer’s instructions.

#### mES cell culture and transfections

E14TG2a mES cells (male genotype, XY) and descended cell lines were cultured on 0.2% gelatin coated plates in 2i/LIF containing medium (1:1 mix of DMEM/F12 (Gibco) and Neurobasal (Gibco) supplemented with 1× Pen-Strep (Gibco), 2 μM Glutamax, 50 μM b-mercaptoethanol (Gibco), 0.1 mM Non-Essential Amino Acids (Gibco), 1 mM Sodium Pyruvate (Gibco), 0.5× N2 Supplement (Gibco), 0.5× B27 Supplement (Gibco), 3 μM GSK3-inhibitor (CHIR99021), 1 μM MEK-inhibitor (PD0325901) and Leukemia Inhibitory Factor (LIF, produced in house)) at 37°C, 5% CO_2_. Cells were passaged every 2–3 days by aspirating medium, dissociated cells with 0.05% Trypsin-EDTA (Gibco) briefly at 37°C before the addition of an equal volume of 1× Trypsin Inhibitor (Sigma) and gentle disruption by pipetting. Cells were pelleted by centrifugation to remove trypsin before resuspending in 2i/LIF medium and plating ∼8×10^4^ cells/ml. Cell lines were transfected with single plasmids using Viafect (Promega) or multiple plasmids using Lipofectamine 3000 (Invitrogen) in 6 well plates according to the manufacturer’s instructions. For antibiotic selection, Blasticidin (BSD) was used at 10 μg/mL, Hygromycin B was used at 100 μg/mL, Genetecin was used at 250 μg/mL. For depletions in mAID-tagged cell lines, 750 μM Indole-3-acetic acid sodium salt (IAA, Sigma) was supplemented to the medium and cells were incubated for the indicated timepoints before harvest. A full list of cell lines used or generated in this study is found in the key resources table.

### Method details

#### CRISPR/Cas9 knock-out/in cells

The generation of *Zfc3h1*^*−/−*^ KO mES cell lines was described in.^47^ CRISPR/Cas9 mediated genomic knock-ins of C-terminal 3xFLAG(3F) mini-AID (mAID) tags were carried out using pGolden (pGCT) homology dependent repair (HDR) donor vectors.^26^^,^^50^ The generation of *Zcchc8-3F-mAID* and *Zfc3h1-3F-mAID* ES cells was described in,^50^ and *Zc3h18-3F-mAID* ES cell lines were generated using the same approach. Single guide RNAs (sgRNAs) were designed using the CHOPCHOP tool (v3)^58^ and cloned into the pSLCas(BB)2A-PURO vector (pX459 Addgene plasmid ID: #48139) as previously described^64^ (Table S3). OsTIR1-expressing ES cells were co-transfected using Lipofectamine 3000 (Thermo) with 2 donor plasmids harboring distinct selection markers (HYG/*NEO*) along with a sgRNA/Cas9 vector in a 1:1:1 ratio. Colonies were maintained under HYG/*NEO* double selection for the donor plasmid markers to increase the likelihood of homozygous knock-in cells. Single cell clones, that survived the selection process, were expanded before screening by western blotting analysis and confirmed by sanger sequencing of the targeted locus.

#### cDNA cloning and exogenous expression of ZFC3H1

Human ZFC3H1 and ZC3H18 cDNA constructs were cloned using full-length cDNA plasmids (pcDNA5-ZFC3H1-3×FLAG,^24^ pcDNA5-ZC3H18-3×FLAG^29^) as a template. Fragments were amplified by Phusion PCR (NEB), using standard conditions, and cloned into DOX-inducible Tet-On (TO) piggyBAC (pB) vectors harboring a C- or N-terminal MYC-tag and Hygromycin B (HYG) selection marker using NEBuilder HiFi DNA assembly (NEB). HeLa Kyoto cells were transfected with pB/TO-Z1^x^-MYC or pB/TO-ZC3H18^x^-MYC vectors along with a pB transposase expressing vector (pBase) and a reverse tetracycline transactivator expressing pB vector containing Zeocin selection marker (pB-rtTA). Cell pools were selected using Hygromycin B and Zeocin until negative control cells no longer survived (7–14 days). For induction of expression constructs, cells were incubated for 1–2 days in culture medium supplemented with 1 μg/mL Doxycycline (Sigma-Aldrich) before harvest. Expression was validated by WB analysis using antibodies against MYC, ZFC3H1 or ZC3H18.

mZFC3H1 cDNA constructs were cloned, using a full-length cDNA plasmid as a template (pCR-XL-TOPO[mZFC3H1], Transomic Technologies), into a piggyBAC (pB) vector containing an N-terminal MYC tag and BSD selection marker using NEBbuilder HiFi DNA assembly (NEB). *Zfc3h1*^*−/−*^ mES cells were transfected with pB-MYC-mZFC3H1^x^-BSD vectors along with a piggyBAC transposase expressing vector (pBase) in a 1:1 ratio using Viafect (Promega). Cell pools were selected with BSD for ∼ 7–10 days or until negative control cells no longer survived. Expression of constructs were validated by western blotting analysis using MYC antibodies. All generated constructs used and generated in this study are listed in the key resources table.

#### RNA isolation and qRT-PCR analysis

Total RNA was isolated using TRIzol reagent (Invitrogen) according to the manufacturer’s instructions. Extracted RNA was treated with TURBO DNase (Invitrogen) according to the manufacturer’s instructions followed by cDNA synthesis from 2 μg RNA using SuperScript III reverse transcriptase (Invitrogen) and a mixture of 80 pmol random primers (Invitrogen) and 20 pmol oligo d(T)_20_VN (Merck). RNA reactions were performed in the presence of Ribolock RNase inhibitor (Thermo). qPCR was performed using Platinum SYBR Green (Invitrogen) and AriaMX Real-Time PCR machine (Agilent Technologies) or ViiA7 Real-Time PCR machine (Applied Biosystems). Primers used for qPCR are listed in Table S3.

#### RNA-sequencing (RNA-seq)

Three biological replicates were prepared for each sample. RNA integrity was assessed using a BioAnalyzer 2000 (Agilent) using RNA Nano chips. Samples were ribodepleted and strand specific libraries were prepared by BGI Tech Solutions (Europe) and sequenced on the DNBseq platform (BGI) (100 bp, paired end).

#### Western blotting analysis

Whole cell protein lysates were prepared using RSB100 buffer (10 mM Tris-HCl pH 7.5, 100 mM NaCl, 2.5 mM MgCl_2_, 0.5% NP-40, 0.5% Triton X-100) freshly supplemented with protease inhibitors (Roche). Samples were denatured by the addition of NuPAGE Loading Buffer (Invitrogen) and NuPAGE Sample Reducing Agent (Invitrogen) before boiling at 95°C for 5 min. SDS-PAGE was carried out on either NuPAGE 4%–12% Bis-Tris or 3%–8% Tris-Acetate (Invitrogen) gels. Western blotting analysis was carried out according to standard protocols with the primary antibodies and HRP- conjugated secondary antibodies listed in the key resources table. Bands were visualized by SuperSignal West Femto ECL substrate (Thermo Scientific) and captured using an Amersham ImageQuant 800 imaging system (GE Healthcare). Images were processed using ImageJ (v1.53).^61^

#### IP experiments

Cells from a confluent 10 cm dish were standardly extracted in HT150 extraction buffer (20 mM HEPES pH 7.4, 150/200 mM NaCl, 0.5% v/v Triton X-100) freshly supplemented with protease inhibitors (Roche). Lysates were sheared by sonication for 3 × 5 s and clarified by centrifugation at 20,000 rcf for 10 min. Cleared lysates were incubated with Pierce anti-*c*-MYC magnetic beads (Thermo Scientific) or ChromoTek GFP-Trap Magnetic Particles M-270 (Proteintech) overnight at 4°C. Beads were washed 3x with HT150 extraction buffer and transferred to a fresh tube for the final wash. Proteins were eluted by incubation at 75°C in NuPAGE Loading Buffer (Invitrogen) for 15 min. Eluates were mixed with NuPAGE Sample Reducing Agent (Invitrogen) and denatured for a further 5 min at 95°C before proceeding with western blotting analysis.

#### Protein expression and purification

Recombinant human ARS2^147−871^, ARS2^147−817(ZnFmut)^, ZFC3H1^11−35(WT)^, ZFC3H1^11−35(MUT),^ ZC3H18^190−211(WT)^ and ZC3H18^190−211(MUT)^ were expressed in BL21 (DE3) *E. coli* cells. ARS2 isoform 4 constructs were cloned from synthetic gene codon optimized for *E. coli* (Invitrogen) as N-terminal 10×His tagged fusion protein cleavable with 3C protease. ZFC3H1 variants were cloned from a synthetic gene codon optimized for *S. frugiperda* (Invitrogen) as N-terminal 6×His-MBP fusion constructs. After *E. coli* cells were grown to OD_600nm_ of 1.2 at 37°C in terrific broth (TB) medium, recombinant protein expression was induced with 0.5 mM IPTG and incubated at 18°C for 16 h. Cultures were harvested by centrifugation at 2000*g* for 15 min, and subsequently bacteria were lysed by sonication in 20 mM HEPES/NaOH pH 7.5, 500 mM NaCl, 20 mM imidazole, 5 mM β-mercaptoethanol, 0.5 mM AEBSF, and 15 U/ml benzonase (Merck). All proteins were initially purified using nickel-based affinity chromatography (IMAC, HIS-Select resin (Sigma-Aldrich)), washed with the wash buffer (lysis buffer supplemented with 1M NaCl, 10 mM MgSO_4_, 50 mM KCl, and 2 mM ATP) and eluted with 20 mM HEPES/NaOH pH 7.5, 100 mM NaCl, 300 mM imidazole, 2 mM DTT. ARS2 variants were subsequently loaded onto 1 mL HiTrap Heparin HP (Cytiva) and gradually eluted with buffer containing 1M NaCl. In the final purification step, ARS2, ZFC3H1 and ZC3H18 eluates were subjected to size exclusion chromatography on a Superdex 75 Increase 10/300 GL column (Cytiva) equilibrated in 20 mM HEPES/NaOH pH 7.5, 100 mM NaCl, 2 mM DTT.

#### *In vitro* pull-down assays

For *in vitro* pull-down assays, ARS2, ZFC3H1 and ZC3H18 variants were mixed at a final concentration of 10 or 30 μM for each protein in pull-down buffer containing 20 mM HEPES/NaOH pH 7.5, 100 mM NaCl, 2 mM DTT, 0.01% Tween 20 at a total volume of 20 μL and incubated on ice for 30 min. The protein mixtures were then incubated with amylose resin (NEB) for 1 h at 4°C. Beads were washed 3x times with pull-down buffer and bound proteins were eluted with SDS loading buffer. Input samples and eluates were analyzed by SDS-PAGE (12.5% gel) and visualized by Coomassie staining.

#### Mass spectrometry

Three biological samples were prepared for each sample. After affinity enrichment, eluted proteins were precipitated on MagResyn HILIC beads in 70% acetonitrile, washed in 95% acetonitrile and 70% ethanol to remove detergents^65^ and digested with trypsin and Lys-C. The resulting peptides were desalted on StageTips and subjected to LCMS analysis on an EASY nano-LC 1200 system (80 min gradient) coupled to an Orbitrap Exploris 480 mass spectrometer (Thermo Scientific). MS data were acquired by data-dependent acquisition and subjected to MaxQuant database searches^66^ using a human Uniprot reviewed sequence database (2021), ‘match between runs’, and ‘label-free Quantification’.

#### Data visualization

Genome browser views based on BigWig files were generated using the R package seqNdisplayR (https://rdrr.io/github/THJlab/seqNdisplayR/). RT-qPCR data was imported from the AriaMx Software (v1.71, Agilent) or QuantStudio Real-time PCR Software (v1.3, Applied Biosystems) and plotted using Graphpad Prism (v9.0.0). Data for multiple sequence alignments was extracted from the Aminode webtool^62^ aligned using Clustal Omega^67^ and displayed using Snapgene (v6.1.1). Volcano plots visualizing the analyzed MS data were generated using custom JavaScript/CSS webtool. Heatmaps were created using the R package Pheatmap (v1.0.12). Displayed data, nascent pA^+/−^ 3′ends,^3^ were centered around the annotated TES and log2 fold change was calculated within 2 nt bins. Displayed TUs represent top 50% of NEXT+PAXT ARS2-sensitive subset extracted after ranking according to mean log_2_fold change of siZCCHC8 relative to ctrl within the displayed region.

### Quantification and statistical analysis

#### Quantification of western blots

Quantification of western blots following immunoprecipitation was performed on non-saturated exposed images using ImageJ (v1.51). Values of co-precipitated proteins were normalized to the bait protein values and plotted relative to control conditions using Graphpad Prism (v9.0.0).

#### RNA-seq data analysis

For all RNAseq data reads were mapped to GRCh38 with HISAT2 (v 2.2.1; ^59^) default settings and the following approach: Genome and splice site information for 'H. sapiens, UCSC hg38 and Refseq gene annotations’ were obtained from the HISAT2 download page (ftp://ftp.ccb.jhu.edu/pub/infphilo/hisat2/data/). Data for canonical chromosomes (i.e., named chr1-22,X and Y but omitting all other sequence contigs) was selected to create a custom index for read mapping. After read mapping only proper read pairs with both reads mapping to the above genome annotation were used for further analysis. Exon read counts overlapping our HeLa in-house annotations^9^ were collected using the featureCounts tools from the subread software suite (v 2.0.1; ^68^) with settings ‘-p -C -s 2 -t exon’. Differentially expressed transcripts were obtained from those raw read counts using R package DESeq2 (v 1.32.0) at a false discovery rate (FDR) cutoff of 0.1. Exon counts for differentially up- and down-regulated transcripts were compared using custom scripts in R. For classification of transcripts into NEXT and PAXT-dependent groups, we used differentially upregulated genes from siRBM7, siZCCHC8, siZFC3H1 -treated HeLa cells^24^ (GSE84172) and similar data from siZC3H3 -treated cells^25^ (GSE131255). Transcripts significantly upregulated (log2FC > 0 and padj <.1) in siRBM7, siZCCHC8, siZFC3H1 and siZC3H3 were selected and used to defined the set of NEXT targets (sig. upregulated in siRBM7 and siZCCHC8, but not sig. upregulated in siZC3H3 and siZFC3H1), PAXT targets (sig. upregulated in siZC3H3 and siZFC3H1, but not sig. upregulated in siRBM7 and siZCCHC8) and NEXT+PAXT targets (upregulated in all 4 knock-downs). ARS2-dependent transcripts were similarly defined as being significantly upregulated (FDR <.1) upon siARS2 knock-down^30^ (GSE99344).

#### Mass spectrometry analysis

Processed LFQ intensities of identified proteins were normalized to the LFQ intensity of the bait protein. Subsequently, Student’s t-test was performed to obtain differential protein enrichment in Z1^1−209(WT)^ IP relative to Z1^1−209(MUT)^ IP.

## Data Availability

• All RNA-seq datasets generated during this study are available at the Gene Expression Omnibus (GEO) under accession code GSE212557. The mass spectrometry proteomics data have been deposited to the ProteomeXchange Consortium via the PRIDE^63^ partner repository with the dataset identifier PXD045842. • This study does not report original code. • Any additional information required to analyze the data in this study is available from the lead contact upon request.

## Supplemental information

Document S1. Figures S1–S5 and Tables S1–S3

Document S2. Article plus supplemental information

## References

[1] Djebali S., Davis C.A., Merkel A., Dobin A., Lassmann T., Mortazavi A., Tanzer A., Lagarde J., Lin W., Schlesinger F. (2012). Landscape of transcription in human cells. Nature.

[2] Steurer B., Janssens R.C., Geverts B., Geijer M.E., Wienholz F., Theil A.F., Chang J., Dealy S., Pothof J., van Cappellen W.A. (2018). Live-cell analysis of endogenous GFP-RPB1 uncovers rapid turnover of initiating and promoter-paused RNA Polymerase II. Proc National Acad Sci.

[3] Wu G., Schmid M., Rib L., Polak P., Meola N., Sandelin A., Jensen T.H. (2020). A Two-Layered Targeting Mechanism Underlies Nuclear RNA Sorting by the Human Exosome. Cell Rep..

[4] Zimmer J.T., Rosa-Mercado N.A., Canzio D., Steitz J.A., Simon M.D. (2021). STL-seq reveals pause-release and termination kinetics for promoter-proximal paused RNA polymerase II transcripts. Mol. Cell.

[5] Almada A.E., Wu X., Kriz A.J., Burge C.B., Sharp P.A. (2013). Promoter directionality is controlled by U1 snRNP and polyadenylation signals. Nature.

[6] Ntini E., Järvelin A.I., Bornholdt J., Chen Y., Boyd M., Jørgensen M., Andersson R., Hoof I., Schein A., Andersen P.R. (2013). Polyadenylation site–induced decay of upstream transcripts enforces promoter directionality. Nat. Struct. Mol. Biol..

[7] Chen Y., Pai A.A., Herudek J., Lubas M., Meola N., Järvelin A.I., Andersson R., Pelechano V., Steinmetz L.M., Jensen T.H., Sandelin A. (2016). Principles for RNA metabolism and alternative transcription initiation within closely spaced promoters. Nat. Genet..

[8] Proudfoot N.J. (2016). Transcriptional termination in mammals: Stopping the RNA polymerase II juggernaut. Science.

[9] Lykke-Andersen S., Žumer K., Molska E.Š., Rouvière J.O., Wu G., Demel C., Schwalb B., Schmid M., Cramer P., Jensen T.H. (2021). Integrator is a genome-wide attenuator of non-productive transcription. Mol. Cell.

[10] Elrod N.D., Henriques T., Huang K.-L., Tatomer D.C., Wilusz J.E., Wagner E.J., Adelman K. (2019). The Integrator Complex Attenuates Promoter-Proximal Transcription at Protein-Coding Genes. Mol. Cell.

[11] Beckedorff F., Blumenthal E., daSilva L.F., Aoi Y., Cingaram P.R., Yue J., Zhang A., Dokaneheifard S., Valencia M.G., Gaidosh G. (2020). The Human Integrator Complex Facilitates Transcriptional Elongation by Endonucleolytic Cleavage of Nascent Transcripts. Cell Rep..

[12] Mendoza-Figueroa M.S., Tatomer D.C., Wilusz J.E. (2020). The Integrator Complex in Transcription and Development. Trends Biochem. Sci..

[13] Austenaa L.M.I., Piccolo V., Russo M., Prosperini E., Polletti S., Polizzese D., Ghisletti S., Barozzi I., Diaferia G.R., Natoli G. (2021). A first exon termination checkpoint preferentially suppresses extragenic transcription. Nat. Struct. Mol. Biol..

[14] Estell C., Davidson L., Steketee P.C., Monier A., West S. (2021). ZC3H4 restricts non-coding transcription in human cells. Elife.

[15] Rouvière J.O., Salerno-Kochan A., Lykke-Andersen S., Garland W., Dou Y., Rathore O., Molska E.Š., Wu G., Schmid M., Bugai A. (2023). ARS2 instructs early transcription termination-coupled RNA decay by recruiting ZC3H4 to nascent transcripts. Mol. Cell.

[16] Estell C., Davidson L., Eaton J.D., Kimura H., Gold V.A.M., West S. (2023). A restrictor complex of ZC3H4, WDR82, and ARS2 integrates with PNUTS to control unproductive transcription. Mol. Cell.

[17] Schmid M., Jensen T.H. (2018). Controlling nuclear RNA levels. Nat. Rev. Genet..

[18] Garland W., Jensen T.H. (2020).

[19] Mitchell P., Petfalski E., Shevchenko A., Mann M., Tollervey D. (1997). The Exosome: A Conserved Eukaryotic RNA Processing Complex Containing Multiple 3′→5′ Exoribonucleases. Cell.

[20] Schuch B., Feigenbutz M., Makino D.L., Falk S., Basquin C., Mitchell P., Conti E. (2014). The exosome-binding factors Rrp6 and Rrp47 form a composite surface for recruiting the Mtr4 helicase. Embo J.

[21] Falk S., Bonneau F., Ebert J., Kögel A., Conti E. (2017). Mpp6 Incorporation in the Nuclear Exosome Contributes to RNA Channeling through the Mtr4 Helicase. Cell Rep..

[22] Wasmuth E.V., Zinder J.C., Zattas D., Das M., Lima C.D. (2017). Structure and reconstitution of yeast Mpp6-nuclear exosome complexes reveals that Mpp6 stimulates RNA decay and recruits the Mtr4 helicase. Elife.

[23] Lubas M., Christensen M.S., Kristiansen M.S., Domanski M., Falkenby L.G., Lykke-Andersen S., Andersen J.S., Dziembowski A., Jensen T.H. (2011). Interaction Profiling Identifies the Human Nuclear Exosome Targeting Complex. Mol. Cell.

[24] Meola N., Domanski M., Karadoulama E., Chen Y., Gentil C., Pultz D., Vitting-Seerup K., Lykke-Andersen S., Andersen J.S., Sandelin A., Jensen T.H. (2016). Identification of a Nuclear Exosome Decay Pathway for Processed Transcripts. Mol. Cell.

[25] Silla T., Schmid M., Dou Y., Garland W., Milek M., Imami K., Johnsen D., Polak P., Andersen J.S., Selbach M. (2020). The human ZC3H3 and RBM26/27 proteins are critical for PAXT-mediated nuclear RNA decay. Nucleic Acids Res..

[26] Gockert M., Schmid M., Jakobsen L., Jens M., Andersen J.S., Jensen T.H. (2022). Rapid factor depletion highlights intricacies of nucleoplasmic RNA degradation. Nucleic Acids Res..

[27] Gerlach P., Garland W., Lingaraju M., Salerno-Kochan A., Bonneau F., Basquin J., Jensen T.H., Conti E. (2022). Structure and regulation of the nuclear exosome targeting complex guides RNA substrates to the exosome. Mol. Cell.

[28] Puno M.R., Lima C.D. (2022). Structural basis for RNA surveillance by the human nuclear exosome targeting (NEXT) complex. Cell.

[29] Andersen P.R., Domanski M., Kristiansen M.S., Storvall H., Ntini E., Verheggen C., Schein A., Bunkenborg J., Poser I., Hallais M. (2013). The human cap-binding complex is functionally connected to the nuclear RNA exosome. Nat. Struct. Mol. Biol..

[30] Iasillo C., Schmid M., Yahia Y., Maqbool M.A., Descostes N., Karadoulama E., Bertrand E., Andrau J.-C., Jensen T.H. (2017). ARS2 is a general suppressor of pervasive transcription. Nucleic Acids Res..

[31] Ogami K., Richard P., Chen Y., Hoque M., Li W., Moresco J.J., Yates J.R., Tian B., Manley J.L. (2017). An Mtr4/ZFC3H1 complex facilitates turnover of unstable nuclear RNAs to prevent their cytoplasmic transport and global translational repression. Gene Dev..

[32] Bresson S.M., Conrad N.K. (2013). The Human Nuclear Poly(A)-Binding Protein Promotes RNA Hyperadenylation and Decay. PLoS Genet..

[33] Gonatopoulos-Pournatzis T., Cowling V.H. (2014). Cap-binding complex (CBC). Biochem. J..

[34] Hallais M., Pontvianne F., Andersen P.R., Clerici M., Lener D., Benbahouche N.E.H., Gostan T., Vandermoere F., Robert M.-C., Cusack S. (2013). CBC–ARS2 stimulates 3′-end maturation of multiple RNA families and favors cap-proximal processing. Nat. Struct. Mol. Biol..

[35] Schulze W.M., Cusack S. (2017). Structural basis for mutually exclusive co-transcriptional nuclear cap-binding complexes with either NELF-E or ARS2. Nat. Commun..

[36] Lykke-Andersen S., Rouvière J.O., Jensen T.H. (2021). ARS2/SRRT: at the nexus of RNA polymerase II transcription, transcript maturation and quality control. Biochem Soc T.

[37] Winczura K., Schmid M., Iasillo C., Molloy K.R., Harder L.M., Andersen J.S., LaCava J., Jensen T.H. (2018). Characterizing ZC3H18, a Multi-domain Protein at the Interface of RNA Production and Destruction Decisions. Cell Rep..

[38] Giacometti S., Benbahouche N.E.H., Domanski M., Robert M.-C., Meola N., Lubas M., Bukenborg J., Andersen J.S., Schulze W.M., Verheggen C. (2017). Mutually Exclusive CBC-Containing Complexes Contribute to RNA Fate. Cell Rep..

[39] Dou Y., Barbosa I., Jiang H., Iasillo C., Molloy K.R., Schulze W.M., Cusack S., Schmid M., Le Hir H., LaCava J., Jensen T.H. (2020). NCBP3 positively impacts mRNA biogenesis. Nucleic Acids Res..

[40] Libri D. (2010). Nuclear Poly(A)-Binding Proteins and Nuclear Degradation: Take the mRNA and Run?. Mol. Cell.

[41] Meola N., Jensen T.H. (2017). Targeting the nuclear RNA exosome: Poly(A) binding proteins enter the stage. RNA Biol..

[42] Silla T., Karadoulama E., Mąkosa D., Lubas M., Jensen T.H. (2018). The RNA Exosome Adaptor ZFC3H1 Functionally Competes with Nuclear Export Activity to Retain Target Transcripts. Cell Rep..

[43] Fan J., Kuai B., Wu G., Wu X., Chi B., Wang L., Wang K., Shi Z., Zhang H., Chen S. (2017). Exosome cofactor hMTR4 competes with export adaptor ALYREF to ensure balanced nuclear RNA pools for degradation and export. Embo J.

[44] Dobrev N., Ahmed Y.L., Sivadas A., Soni K., Fischer T., Sinning I. (2021). The zinc-finger protein Red1 orchestrates MTREC submodules and binds the Mtl1 helicase arch domain. Nat. Commun..

[45] Foucher A.-E., Touat-Todeschini L., Juarez-Martinez A.B., Rakitch A., Laroussi H., Karczewski C., Acajjaoui S., Soler-López M., Cusack S., Mackereth C.D. (2022). Structural analysis of Red1 as a conserved scaffold of the RNA-targeting MTREC/PAXT complex. Nat. Commun..

[46] Wang Y., Fan J., Wang J., Zhu Y., Xu L., Tong D., Cheng H. (2021). ZFC3H1 prevents RNA trafficking into nuclear speckles through condensation. Nucleic Acids Res..

[47] Garland W., Comet I., Wu M., Radzisheuskaya A., Rib L., Vitting-Seerup K., Lloret-Llinares M., Sandelin A., Helin K., Jensen T.H. (2019). A Functional Link between Nuclear RNA Decay and Transcriptional Control Mediated by the Polycomb Repressive Complex 2. Cell Rep..

[48] Melko M., Winczura K., Rouvière J.O., Oborská-Oplová M., Andersen P.K., Heick Jensen T. (2020). Mapping domains of ARS2 critical for its RNA decay capacity. Nucleic Acids Res..

[49] Natsume T., Kanemaki M.T. (2017). Conditional Degrons for Controlling Protein Expression at the Protein Level. Annu. Rev. Genet..

[50] Garland W., Müller I., Wu M., Schmid M., Imamura K., Rib L., Sandelin A., Helin K., Jensen T.H. (2022). Chromatin modifier HUSH co-operates with RNA decay factor NEXT to restrict transposable element expression. Mol. Cell.

[51] Schulze W.M., Stein F., Rettel M., Nanao M., Cusack S. (2018). Structural analysis of human ARS2 as a platform for co-transcriptional RNA sorting. Nat. Commun..

[52] Hein M.Y., Hubner N.C., Poser I., Cox J., Nagaraj N., Toyoda Y., Gak I.A., Weisswange I., Mansfeld J., Buchholz F. (2015). A Human Interactome in Three Quantitative Dimensions Organized by Stoichiometries and Abundances. Cell.

[53] Wu G., Schmid M., Jensen T.H. (2021). 3′ End sequencing of pA+ and pA− RNAs. Methods Enzymol..

[54] Fan J., Kuai B., Wang K., Wang L., Wang Y., Wu X., Chi B., Li G., Cheng H. (2018). mRNAs are sorted for export or degradation before passing through nuclear speckles. Nucleic Acids Res..

[55] Zhou Y., Zhu J., Schermann G., Ohle C., Bendrin K., Sugioka-Sugiyama R., Sugiyama T., Fischer T. (2015). The fission yeast MTREC complex targets CUTs and unspliced pre-mRNAs to the nuclear exosome. Nat. Commun..

[56] Gruber J.J., Olejniczak S.H., Yong J., La Rocca G., Dreyfuss G., Thompson C.B. (2012). Ars2 Promotes Proper Replication-Dependent Histone mRNA 3′ End Formation. Mol. Cell.

[57] O’Sullivan C., Christie J., Pienaar M., Gambling J., Nickerson P.E.B., Alford S.C., Chow R.L., Howard P.L. (2015). Mutagenesis of ARS2 Domains To Assess Possible Roles in Cell Cycle Progression and MicroRNA and Replication-Dependent Histone mRNA Biogenesis. Mol. Cell Biol..

[58] Labun K., Montague T.G., Krause M., Torres Cleuren Y.N., Tjeldnes H., Valen E. (2019). CHOPCHOP v3: expanding the CRISPR web toolbox beyond genome editing. Nucleic Acids Res..

[59] Kim D., Langmead B., Salzberg S.L. (2015). HISAT: a fast spliced aligner with low memory requirements. Nat. Methods.

[60] Love M.I., Huber W., Anders S. (2014). Moderated estimation of fold change and dispersion for RNA-seq data with DESeq2. Genome Biol.

[61] Schneider C.A., Rasband W.S., Eliceiri K.W. (2012). NIH Image to ImageJ: 25 years of image analysis. Nat. Methods.

[62] Chang K.T., Guo J., di Ronza A., Sardiello M. (2018). Aminode: Identification of Evolutionary Constraints in the Human Proteome. Sci. Rep..

[63] Perez-Riverol Y., Bai J., Bandla C., García-Seisdedos D., Hewapathirana S., Kamatchinathan S., Kundu D.J., Prakash A., Frericks-Zipper A., Eisenacher M. (2022). The PRIDE database resources in 2022: a hub for mass spectrometry-based proteomics evidences. Nucleic Acids Res..

[64] Ran F.A., Hsu P.D., Lin C.-Y., Gootenberg J.S., Konermann S., Trevino A.E., Scott D.A., Inoue A., Matoba S., Zhang Y., Zhang F. (2013). Double Nicking by RNA-Guided CRISPR Cas9 for Enhanced Genome Editing Specificity. Cell.

[65] Batth T.S., Tollenaere M.A.X., Rüther P., Gonzalez-Franquesa A., Prabhakar B.S., Bekker-Jensen S., Deshmukh A.S., Olsen J.V. (2019). Protein Aggregation Capture on Microparticles Enables Multipurpose Proteomics Sample Preparation. Mol. Cell. Proteomics.

[66] Cox J., Mann M. (2008). MaxQuant enables high peptide identification rates, individualized p.p.b.-range mass accuracies and proteome-wide protein quantification. Nat. Biotechnol..

[67] Sievers F., Wilm A., Dineen D., Gibson T.J., Karplus K., Li W., Lopez R., McWilliam H., Remmert M., Söding J. (2011). Fast, scalable generation of high-quality protein multiple sequence alignments using Clustal Omega. Mol. Syst. Biol..

[68] Liao Y., Smyth G.K., Shi W. (2014). featureCounts: an efficient general purpose program for assigning sequence reads to genomic features. Bioinformatics.

