# Supplementary material for: Dual agonistic and antagonistic roles of ZC3H18 provide for co-activation of distinct nuclear RNA decay pathways
Source: Cell Rep. 2023 Oct 26;42(11):113325. doi: 10.1016/j.celrep.2023.113325 (PMC10720265; doi:10.1016/j.celrep.2023.113325)
Supplement: Document S1. Figures S1–S5 and Tables S1–S3 [file mmc1.pdf]

Cell Reports, Volume *42*

Supplemental information

Dual agonistic and antagonistic roles of ZC3H18 provide for co-activation of distinct nuclear RNA decay pathways

Patrik Polák, William Garland, Om Rathore, Manfred Schmid, Anna Salerno-Kochan, Lis Jakobsen, Maria Gockert, Piotr Gerlach, Toomas Silla, Jens S. Andersen, Elena Conti, and Torben Heick Jensen

Supplemental Figure 1; Related to Figure 1

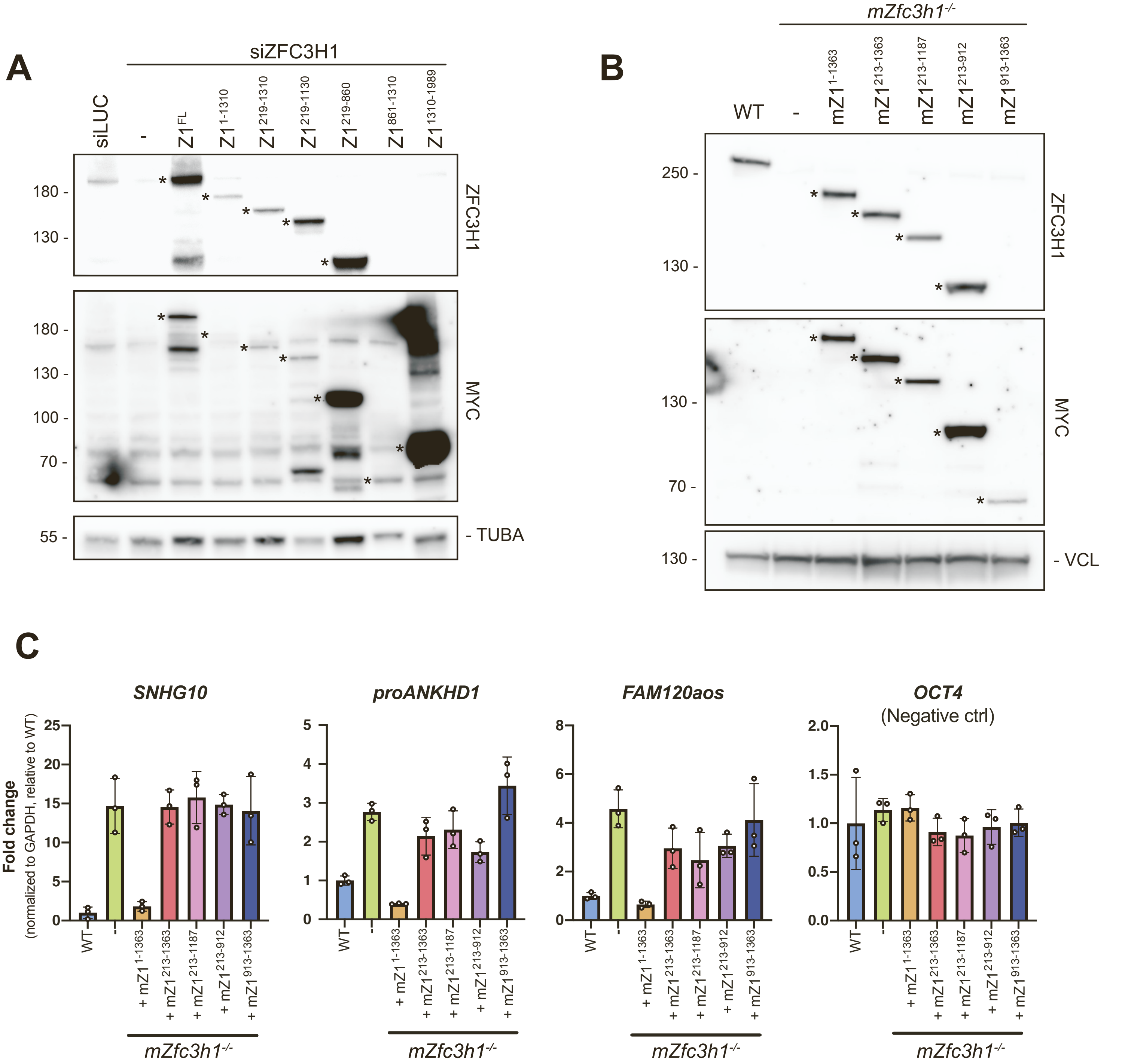

Supplemental Figure 1, related to Figure 1: The ZFC3H1 N-terminus harbors important information for its function in RNA decay.

(A) WB analysis showing expression in siZFC3H1 cells of the generated ZFC3H1 variants from Figure 1C. Membranes were probed with antibodies against ZFC3H1, MYC and Tubulin alpha (TUBA) as a loading control. $Z1^{FL}$, $Z1^{1-1310}$, $Z1^{219-1310}$, $Z1^{219-1130}$ and $Z1^{219-860}$ variants were detectable with anti-ZFC3H1 antibody, while $Z1^{861-1310}$ and $Z1^{1311-1989}$ variants could only be detected with the anti-MYC antibody. Migrations of Z1 bands are indicated by asterisks. Migration of protein markers is indicated to the left. A siLUC sample was added as a control. **(B)** WB analysis showing expression of the generated mouse ZFC3H1 variants in *mZfc3h1*$^{-/-}$ mES cells. Membranes were probed with antibodies against ZFC3H1, MYC and Vinculin (VCL) as a loading control. Migrations of mZ1 bands are indicated by asterisks. WT mES cells were added as a control. **(C)** qRT-PCR analysis of selected mouse PAXT substrates and OCT4 mRNA (negative control) from total RNA isolated from cells used in (B). RT primers as in Figure 1C. qPCR amplicons were positioned in TU 5'end regions and for SNHG10 and OCT4 amplicons were spanning the first exon-exon junction. Results were normalized to GAPDH mRNA levels and plotted as fold changes relative to WT control samples. Data representation as in Figure 1C.

Supplemental Figure 2; Related to Figure 2

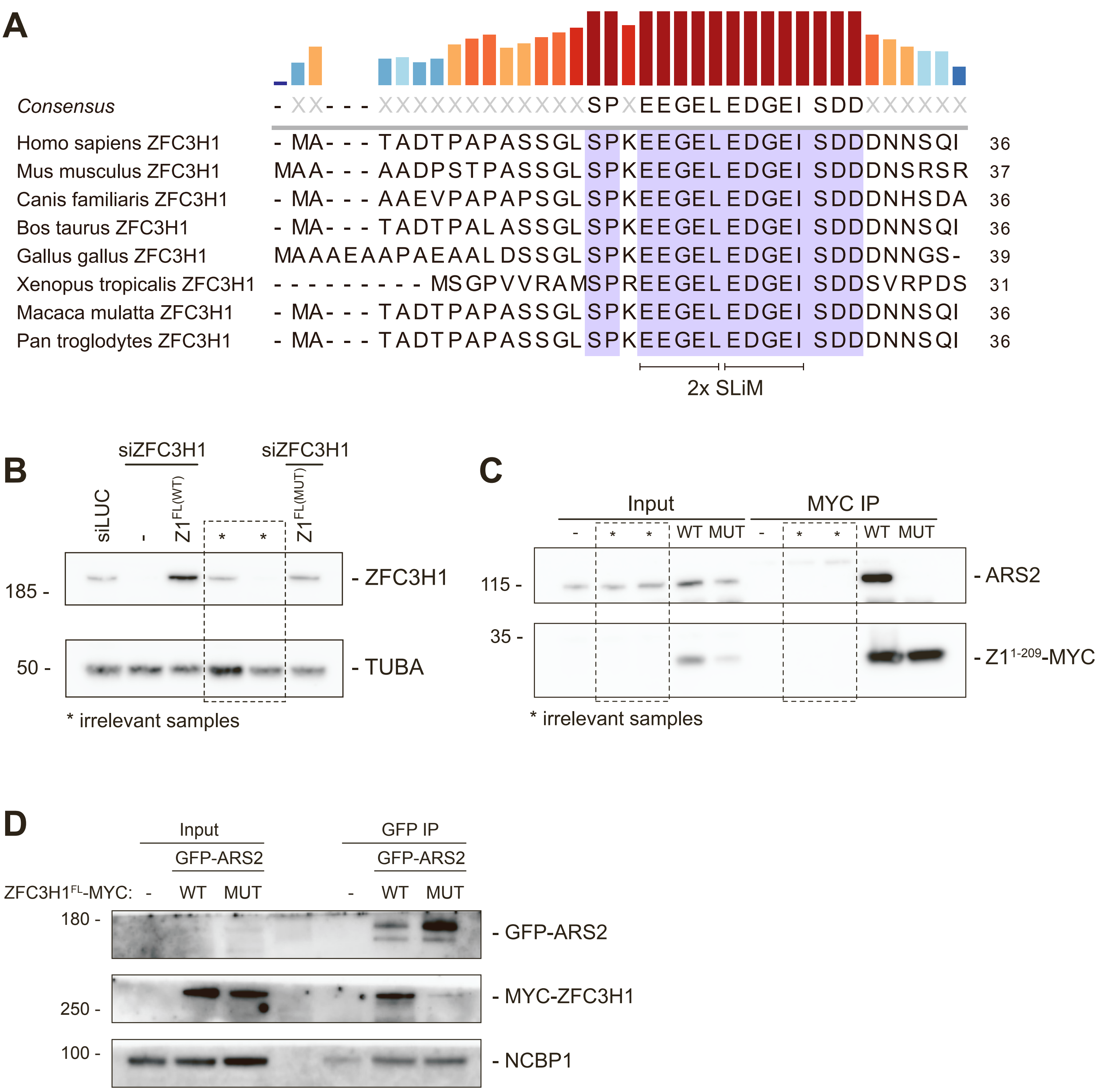

Supplemental Figure 2, related to Figure 2: A conserved short linear motif connects ZFC3H1 to the CBC via ARS2.

(A) Multiple sequence alignment analysis of ZFC3H1 protein sequences from selected species, showing N-terminal conservation. Two copies of a conserved SLiM (EEGEL and EDGEI) are underlined. The height and colour of the bars indicate the level of conservation for each amino acid position according to the colour gradient displayed in top right. **(B)** Uncropped WB membranes of the top right panel of Fig. 2A. Deleted lanes holding irrelevant samples are highlighted by a dashed box. **(C)** Uncropped WB membranes of Fig. 2C. Layout as in (B). **(D)** WB analysis of GFP IPs from lysates of either WT HeLa cells or MYC-tagged ZFC3H1^{WT} and MYC-tagged ZFC3H1^{MUT} cells transiently expressing GFP-ARS2. Input and IP samples were probed with antibodies against GFP, MYC and NCBP1 as a loading and IP control.

Supplemental Figure 3; Related to Figure 3

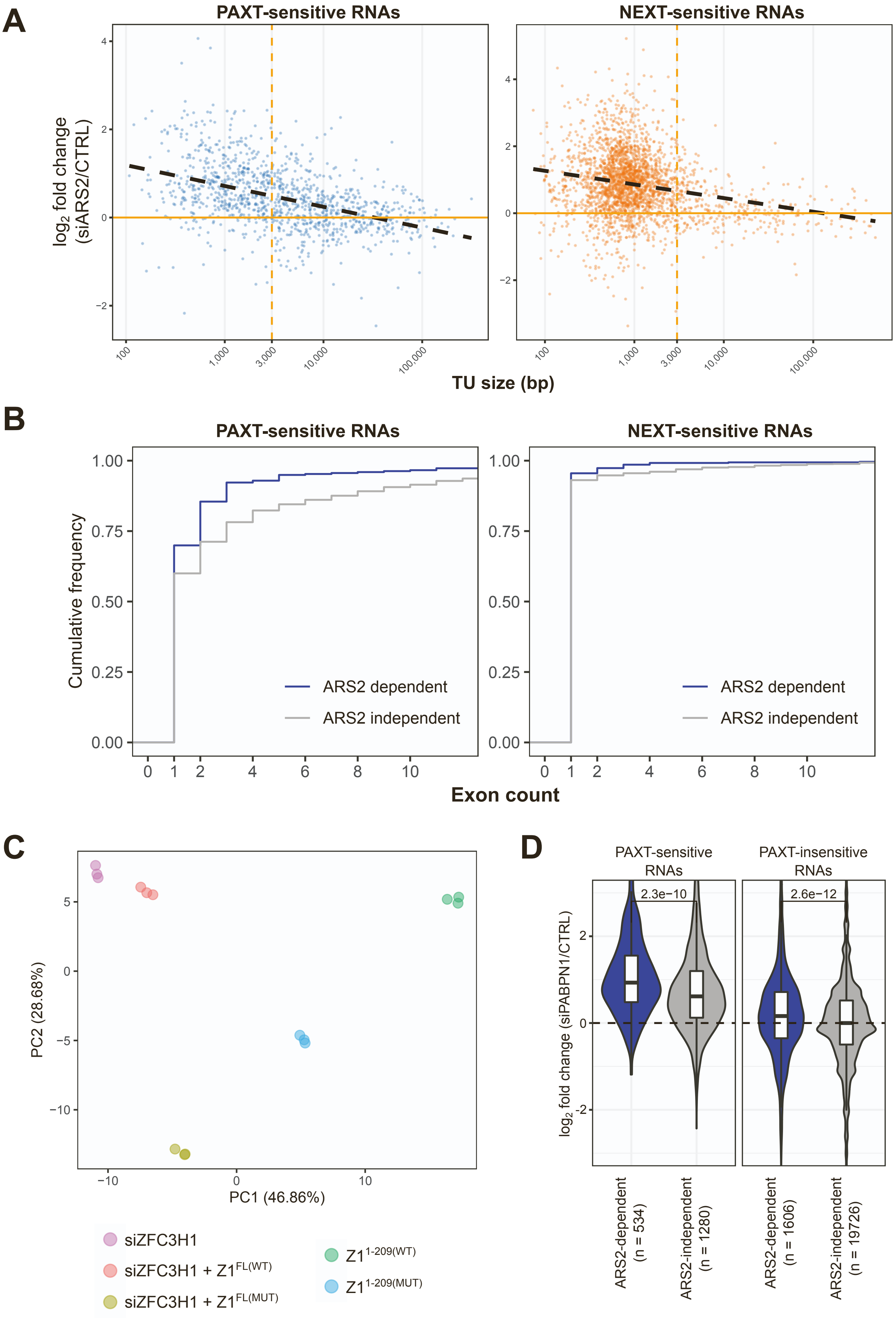

Supplemental Figure 3, related to Figure 3: The ZFC3H1-ARS2 connection facilitates the decay of short, adenylated transcripts.

(A) Scatter plots showing distributions of log_2 fold RNA changes in total RNA-seq datasets generated from siARS2-treated HeLa cells and their controls (CTRL)[30]. Data were stratified by TU size (x-axis) and PAXT- (left) or NEXT- (right) sensitivity[3]. **(B)** Cumulative frequency plots showing distributions of exon counts among ARS2-dependent (blue) and -independent (grey) RNAs, stratified by their PAXT- (left) and NEXT- (right) sensitivity as in (A). **(C)** Principal component analysis showing clustering of the three replicates of RNA-seq samples used in Figure 3C-D (siZFC3H1, siZFC3H1 + $Z1^{FL(WT)}$, siZFC3H1 + $Z1^{FL(MUT)}$) and Figure 5F ($Z1^{1\text{-}209(WT)}$, $Z1^{1\text{-}209(MUT)}$). **(D)** Violin box plots as in Fig. 3A, but showing total RNA-seq datasets generated from siPABPN1-treated HeLa cells[24]. Transcripts exhibiting NEXT-sensitivity were included in all categories as NEXT-mediated RNA decay was not disrupted in these samples. P-values were calculated as in Fig. 3A.

Supplemental Figure 4; Related to Figure 4

A

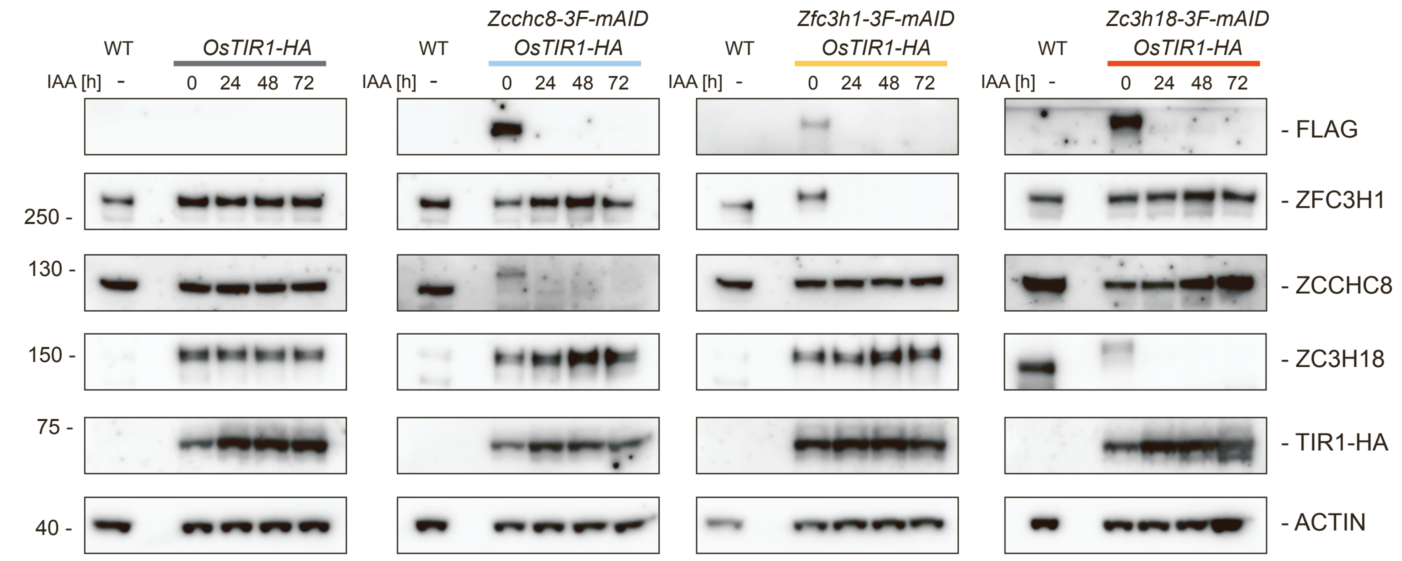

B

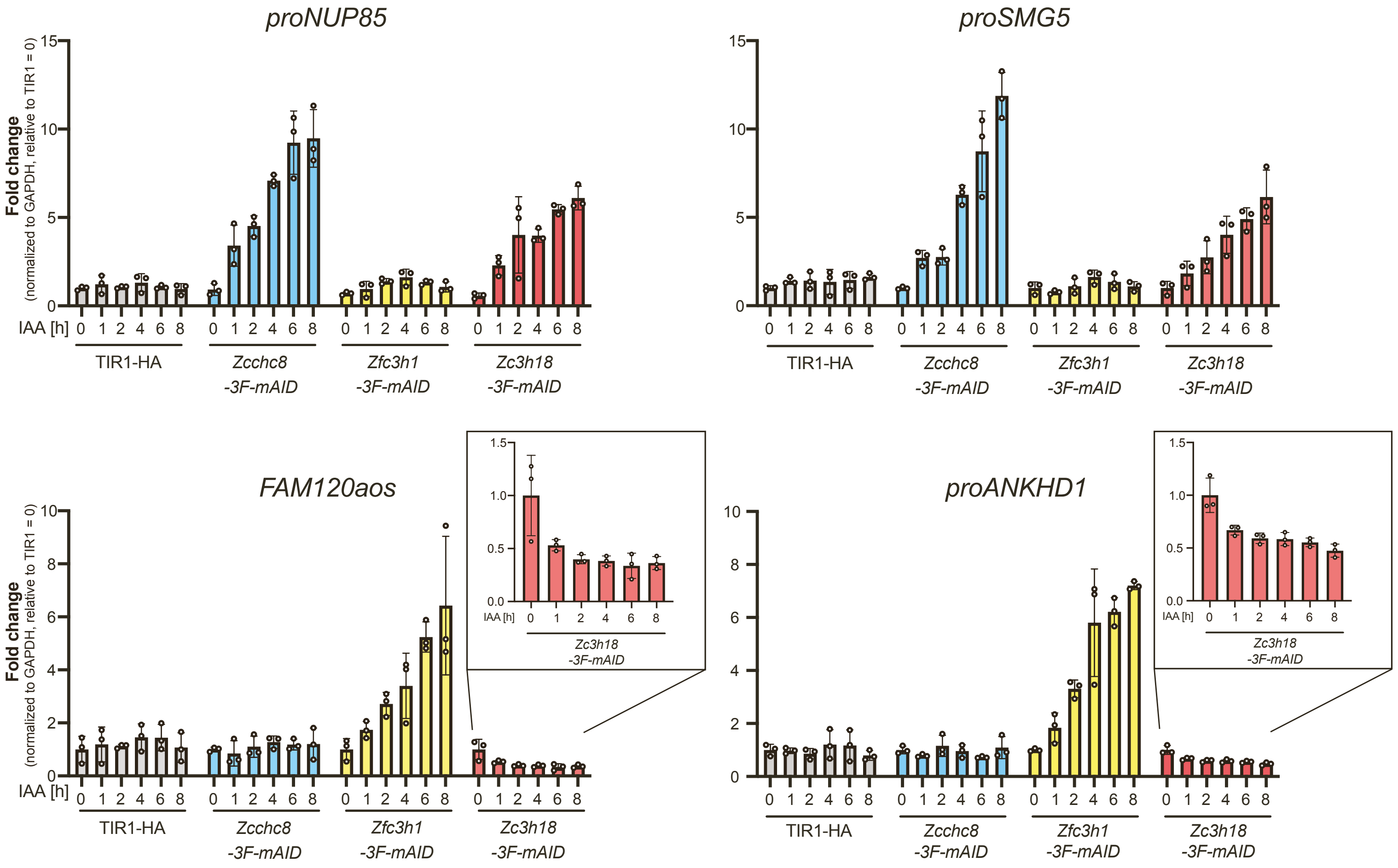

Supplemental Figure 4, related to Figure 4: ZC3H18 antagonizes the ARS2-dependent PAXT pathway.

(A) WB analysis showing time course depletion experiments of 3F-mAID-tagged ZCCHC8, ZFC3H1 and ZC3H18 fusion proteins in mES cells, expressing OsTIR1-HA following 0, 24, 48, or 72 h of IAA treatment as indicated. Lysates from WT mES cells and from IAA time series of the parental cell line (left panel) were included as controls. Membranes were probed against FLAG, ZFC3H1, ZCCHC8, ZC3H18, HA and ACTIN as a loading control. **(B)** qRT-PCR analysis of two NEXT (top) and two PAXT (bottom) substrates from total RNA isolated from *Zcchc8-3F-mAID*, *Zfc3h1-3F-mAID*, *Zc3h18-3F-mAID* and control *OsTIR-HA* mES cells following 0 to 8 h of IAA treatment. Results were normalized to GAPDH mRNA levels and plotted as fold change relative to *OsTIR-HA* control samples at the 0 h time point. Separate plots with adjusted scales are additionally displayed for PAXT substrate levels in *Zc3h18-3F-mAID* samples for better visualization. Data representation as in Fig. 1C.

Supplemental Figure 5; Related to Figure 5

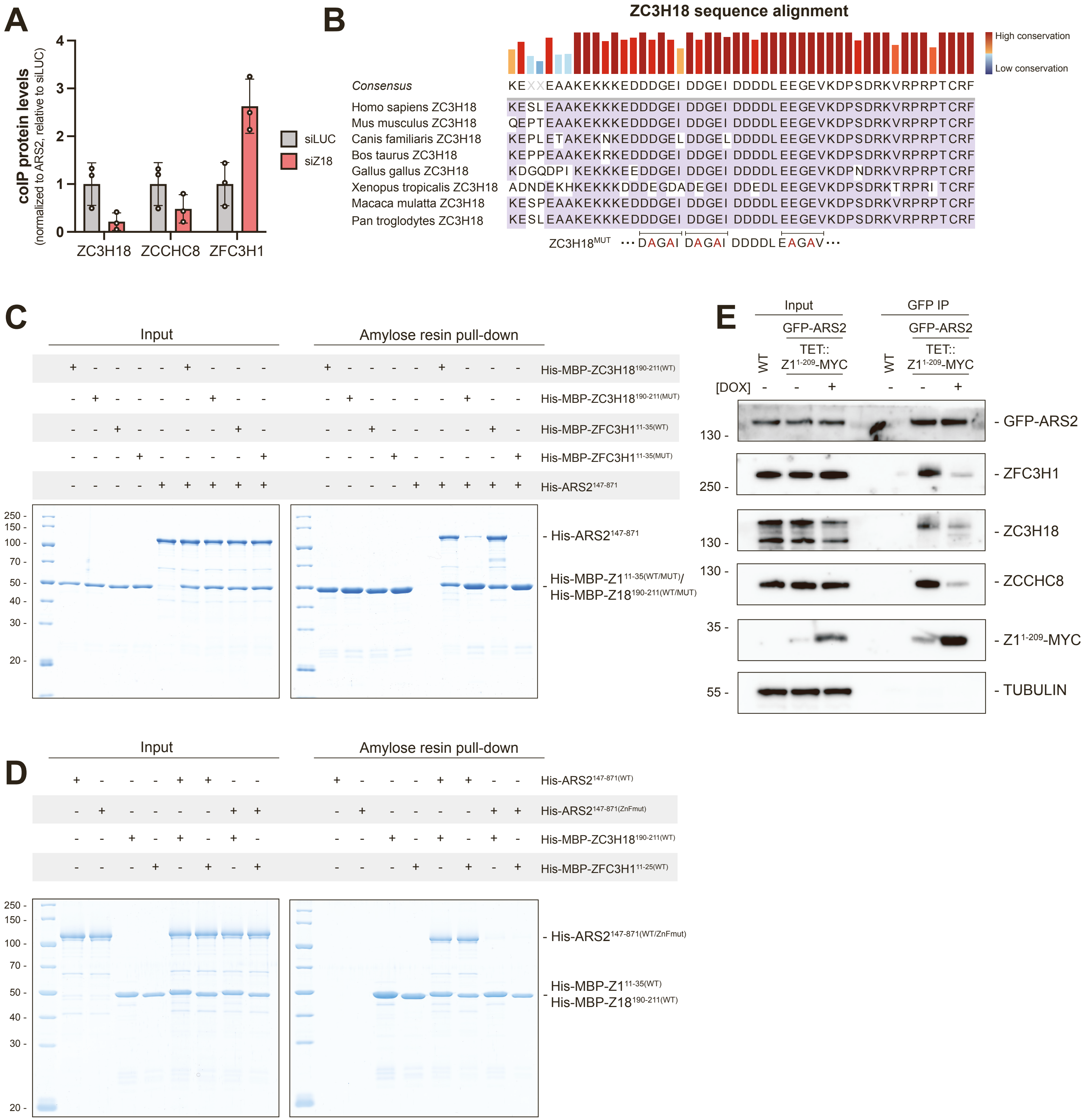

Supplemental Figure 5, related to Figure 5: ZFC3H1 and ZC3H18 compete for ARS2 binding.

(A) Quantification of WB analysis from Fig. 5A and including 2 additional replicates. ZC3H18, ZCCHC8 and ZFC3H1 values from IP samples were normalized to MYC-ARS2 values from the respective IPs. Values from siZ18 conditions were plotted relative to siLUC control conditions. Individual points indicate replicate samples. **(B)** Multiple sequence alignment analysis of ZC3H18 protein sequences from selected species showing three copies of a conserved and underlined SLiM (DDGEI, DDGEI and EDGEV) similar to the SLiM found in the ZFC3H1 N-terminus (Figure S2). Mutations in the conserved SLiM motifs (ZC3H18^{mut}) are indicated below the schematic. Levels of conservation are indicated as in Figure S2. **(C)** Coomassie stained SDS-PAGE gels showing input and Amylose resin pull-down samples of His-ARS2$^{147\text{-}871}$ incubated with either WT or MUT variants of His-MBP-Z1$^{1\text{-}35}$ and His-MBP-Z18$^{190\text{-}211}$. '+' on top indicates which proteins were added. Protein markers are indicated to the left. **(D)** Coomassie stained SDS-PAGE gels showing input and Amylose resin pull-down samples of His-MBP-Z1$^{1\text{-}35}$ or His MBP-Z18$^{190\text{-}211}$ incubated with either WT or ZnFmut (K698A, K719A, K743A) variants of His-ARS1$^{147\text{-}871}$. '+' on top indicates which proteins were added. Protein markers are indicated to the left. **(E)** WB analysis of GFP IPs from lysates of HeLa cells stably integrated with DOX-inducible MYC-Z1$^{1\text{-}209}$, transiently transfected with GFP-ARS2 and -/+ DOX treatment. WT HeLa cells were included as negative IP controls. Input and IP samples were probed with GFP, ZFC3H1, ZC3H18, ZCCHC8, MYC and TUBULIN was used as a loading control.

Supplemental Tables, related to STAR methods

Table S1: siRNAs used during this study

Name	Sequence (sense/antisense)
siZFC3H1-5'UTR	GGGCUAAGGUUGUGUGGAAdTdT/UUCCACACAACCUUAGCC CdTdT
siZFC3H1-3'UTR	GGUCAAAUAUUAUGUGCAAdTdT/UUGCACAUAAUAUUUGAC CdTdT
siLUC	CUUACGCUGAGUACUUCGAdTdT/UCGAAGUACUCAGCGUAA GdTdT
siZC3H18	GGAAUGAAUUGUAGGUUUAdTdT/UAAACCUACAAUUCAUUC CdTdT

Table S2: sgRNAs used during this study

Name	Sequence
hZC3H18_CRISPR_KI_F	CACCGAGCATAGGCCGTGCCCCGAC
hZC3H18_CRISPR_KI_F	AAACGTCGGGGCACGGCCTATGCTC
mZC3H18_CRISPR_KI_F	AGATCCCCGGGAAAGTGTAG
mZC3H18_CRISPR_KI_F	CTACACTTTCCCGGGGATCT

Table S3: qPCR primers used during this study

Species	Name	Sequence
Human	proASH1L F	TAGGGAGTGAGGCCAGTAGGA
Human	proASH1L R	TCCCAGGTTGGCAACTCAAC
Human	proDDX6 F	CACACCGACGAGAAAAGTTCG
Human	proDDX6 R	CATTTCTCAATCACGTCGCGG
Human	proDHCR7 F	GCGACGCACATTGATGGAG
Human	proDHCR7 R	GCCCTAGGACCAAGAACTGTG
Human	proPRKAA1 F	AGGGGCTTCCTGGAACGATTA
Human	proPRKAA1 R	GACCGCAGAGTGGTAGTTAGG
Human	proPRKACB F	GCTGAACAGTATTCCGCGAC
Human	proPRKACB R	AGTGGGAACCGCTAGCTTTG
Human	proSAMD4B F	CTTAGCGCTTTCTGTGCATTCT
Human	proSAMD4B R	CTCTCCGATGGGAATGGCGA
Human	GAPDH F	GAGTCAACGGATTTGGTCGT
Human	GAPDH R	TGAGGTCAATGAAGGGGTCA
Human	18S rRNA F	GCAATTATTCCCCATGAACG
Human	18S rRNA R	GGGACTTAATCAACGCAAGC
Human	FOXD2-AS1 F	CTTTCTCCACCCGGATCTTC

Human	FOXD2-AS1 R	TGGGGAACATGTCTGAGGTC
Human	proSTAT3 F	CCGCCTGCTTTGAACTTCAG
Human	proSTAT3 R	TCTCCCCACGCACTCTAGTA
Human	proKLF6 F	AAGTTTTAGAGGGTCCGGCA
Human	proKLF6 R	CTCTGCATAACCTTCACCG
Human	proLY6K F	AATCCACCTTGTGCCCTCTG
Human	proLY6K R	ACCTCTGGACCAGCCATTTG
Human	proDIP2A F	AACTGCATGGGAATGCTTGC
Human	proDIP2A R	TTCCAGCTGGCAAAACTTGG
Human	ZNF250 F	TAGAGGCGACTCGGTGCTAT
Human	ZNF250 R	AGGCCTACTATGGCTCACAA
Human	proEXT1 F	TCTAATGGCTGCAGGGAAAC
Human	proEXT1 R	TAGCTGGGACAGTTGGCAAT
Human	proDNAJB4 F	TTTCTGGCGTTTCTGATTGA
Human	proDNAJB4 R	ACCAAAACGCAGGTTGTTTA
Mouse	SNHG10 F	GCCTTCCATGCCTCACCG
Mouse	SNHG10 R	GAATCAGAGGATCCTGCAAG
Mouse	proANKHD1 F	CTTGGTCCTGAGAACTGTCCG
Mouse	proANKHD1 R	CGCTCCGAGGTCACTCAACA
Mouse	FAM120aos F	CAGGAGTGACCCATCCATCG
Mouse	FAM120aos R	CCATCAAGGCCCTAAGCGC
Mouse	OCT4 F	CAGCAGATCACTCACATCGCCA
Mouse	OCT4 R	GCCTCATACTCTTCTCGTTGGG
Mouse	proNUP85 F	GCGGTAGTTGTGATCCCTCCC
Mouse	proNUP85 R	GGCTGGTTCTGAACTCCCGA
Mouse	proSMG5 F	CTGAGCGTTCTAGCGCAGG
Mouse	proSMG5 R	TTAGCCGCTGAGTCATCTCG
